# B cell-derived anti-beta 2 glycoprotein I antibody mediates hyperhomocysteinemia-aggravated hypertensive glomerular lesions by triggering ferroptosis

**DOI:** 10.1038/s41392-023-01313-x

**Published:** 2023-03-13

**Authors:** Xing Du, Xiaolong Ma, Ying Tan, Fangyu Shao, Chun Li, Yang Zhao, Yutong Miao, Lulu Han, Guohui Dang, Yuwei Song, Dongmin Yang, Zhenling Deng, Yue Wang, Changtao Jiang, Wei Kong, Juan Feng, Xian Wang

**Affiliations:** 1 Department of Physiology and Pathophysiology, School of Basic Medical Sciences, Key Laboratory of Molecular Cardiovascular Science, Ministry of Education, Peking University, 100191 Beijing, P. R. China; 2 Department of Nephrology, Peking University First Hospital, 100034 Beijing, P. R. China; 3 Department of Rheumatology and Immunology, Peking University People’s Hospital, Beijing, China; 4 Department of Laboratory Medicine, Peking University Third Hospital, 100083 Beijing, P. R. China; 5 Department of Nephrology, Peking University Third Hospital, 100083 Beijing, P. R. China

**Keywords:** Immunological disorders, Kidney diseases

## Abstract

Hyperhomocysteinemia (HHcy) is a risk factor for chronic kidney diseases (CKDs) that affects about 85% CKD patients. HHcy stimulates B cells to secrete pathological antibodies, although it is unknown whether this pathway mediates kidney injury. In HHcy-treated 2-kidney, 1-clip (2K1C) hypertensive murine model, HHcy-activated B cells secreted anti-beta 2 glycoprotein I (β_2_GPI) antibodies that deposited in glomerular endothelial cells (GECs), exacerbating glomerulosclerosis and reducing renal function. Mechanistically, HHcy 2K1C mice increased phosphatidylethanolamine (PE) (18:0/20:4, 18:0/22:6, 16:0/20:4) in kidney tissue, as determined by lipidomics. GECs oxidative lipidomics validated the increase of oxidized phospholipids upon Hcy-activated B cells culture medium (Hcy-B CM) treatment, including PE (18:0/20:4 + 3[O], PE (18:0a/22:4 + 1[O], PE (18:0/22:4 + 2[O] and PE (18:0/22:4 + 3[O]). PE synthases ethanolamine kinase 2 (etnk2) and ethanolamine-phosphate cytidylyltransferase 2 (pcyt2) were increased in the kidney GECs of HHcy 2K1C mice and facilitated polyunsaturated PE synthesis to act as lipid peroxidation substrates. In HHcy 2K1C mice and Hcy-B CM-treated GECs, the oxidative environment induced by iron accumulation and the insufficient clearance of lipid peroxides caused by transferrin receptor (TFR) elevation and down-regulation of SLC7A11/glutathione peroxidase 4 (GPX4) contributed to GECs ferroptosis of the kidneys. In vivo, pharmacological depletion of B cells or inhibition of ferroptosis mitigated the HHcy-aggravated hypertensive renal injury. Consequently, our findings uncovered a novel mechanism by which B cell-derived pathogenic anti-β_2_GPI IgG generated by HHcy exacerbated hypertensive kidney damage by inducing GECs ferroptosis. Targeting B cells or ferroptosis may be viable therapeutic strategies for ameliorating lipid peroxidative renal injury in HHcy patients with hypertensive nephropathy.

## Introduction

Homocysteine (Hcy) is an intermediate aminothiol derived from methionine catabolism. Generally, a high plasma Hcy level (>15 μM), known as hyperhomocysteinemia (HHcy), is widespread in Asians due to dietary patterns and genetic factors.^1,2^ Especially in patients with chronic kidney disease (CKD), the proportion of HHcy is as high as 85%, while it is only 5–7% in the general population.^3–5^ HHcy has been identified as a risk factor of CKD and contributes to cardiovascular complications.^6–9^ A cross-sectional survey reveals that hypertension is a major cause of CKD,^10^ but the molecular mechanisms by which HHcy mediates hypertension-associated kidney damage remains poorly understood.

Immune system disorders contribute to the progression of kidney disease.^11,12^ Lymphocytic infiltration was identified in the renal interstitial spaces adjacent to damaged glomeruli and tubules in patients with hypertensive renal damage.^13^ And clinical studies suggest that B cell activation and IgG production is involved in the pathogenesis of hypertension and end organ damage.^14,15^ Our laboratory has innovatively reported that Hcy promotes B cell proliferation and antibody (Ab) secretion by up-regulating glycolytic metabolism.^16,17^ Importantly, we noted that human and mouse plasma anti-beta 2 glycoprotein I (β_2_GPI) antibody levels are also significantly increased by HHcy, which exacerbates abdominal aortic aneurysm (AAA) progression.^18^ Kidney damage is a well-recognized complication of the antiphospholipid syndrome (APS), which is a systemic autoimmune disease defined by thrombotic or obstetrical events that occur in patients with persistent antiphospholipid antibodies (aPL).^19,20^ Anti-β_2_GPI antibodies, one of the aPLs, are associated with an increased risk of kidney diseases, and B cell depletion with CD20 monoclonal antibodies (mAbs) is effective in restoring the aPL-related decline in renal function.^21,22^ Therefore, we hypothesized that B cell-derived anti-β_2_GPI IgG induced by HHcy might be involved in the development of hypertensive renal injury.

Ferroptosis is a newly discovered metabolic cell death driven by iron-dependent lipid peroxidation, and characterized by accumulation of redox-active iron, loss of antioxidant capacity, and peroxidation of phospholipid-containing polyunsaturated fatty acyl tails (PL-PUFAs).^23–25^ Excess iron catalyzes reactive oxygen species (ROS) production via the Fenton reaction and attacks unsaturated membrane phospholipids.^26–28^ SLC7A11/xCT and glutathione peroxidase 4 (GPX4), as key components of elimination lipid peroxides, can protect cell from ferroptosis.^29,30^ Ferroptosis mediates the progression of multiple kidney diseases, including ischemia-reperfusion injury and diabetic nephropathy.^31,32^ It is consistent with previous reports that abnormal lipid metabolism is not only a clinical manifestation of kidney disease, but also an important pathogenic factor.^33,34^ We have reported that HHcy increases the anabolism and catabolism of lipids in lymphocytes, macrophages and adipocytes.^35–37^ However, whether HHcy-induced anti-β_2_GPI antibodies mediate renal injury by triggering ferroptosis needs to be investigated.

The present study showed that B-cell-derived anti-β_2_GPI IgG production and deposition at glomerular endothelial cells (GECs) in HHcy hypertensive renal injury mice triggered ferroptosis and glomerulosclerosis. The anti-CD20 mAb and Fer-1, a ferroptosis inhibitor, could effectively ameliorate HHcy-aggravated hypertensive renal injury. This finding provides a novel mechanistic explanation for disease progression in the CKD population with HHcy and potential targets for intervention in HHcy-associated renal injury.

## Results

### HHcy aggravates hypertensive kidney damage mediated by B cell-derived anti-β_2_GPI IgG

**Fig. 1:**
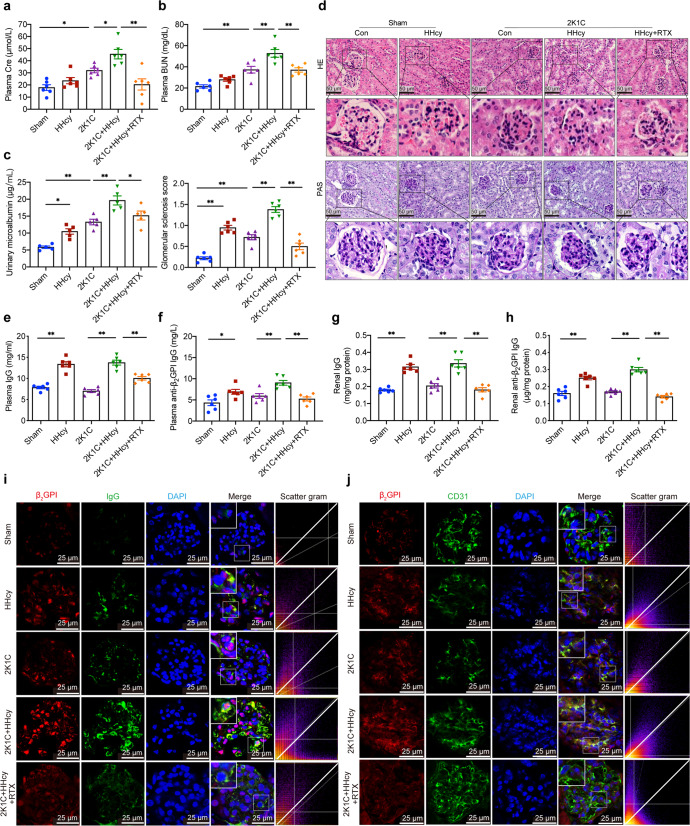
HHcy aggravates hypertensive kidney damage mediated by B cell-derived anti-β2GPI IgG. C57BL/6J mice (8 weeks old) treated with sham or 2K1C surgery were given drinking water with or without Hcy (1.8 g/L) for 4 weeks. To assess the role of B cells in renal injury, Rituximab was administered to HHcy 2K1C mice at the start of modeling (i.p. 75 μg/20 g body weights every other day for 4 weeks). Plasma Cre (**a**), BUN (**b**), and urinary microalbumin (**c**) were assayed to evaluate renal function using ELISA. **d** Representative histochemical staining of kidney paraffin sections with hematoxylin and eosin (HE, top) and periodic acid-Schiff (PAS, bottom) (scale bar, 50 μm). Quantification of the glomerular matrix index was performed for each group (0 = normal glomeruli, 1 = thickening of the GBM, 1.5 = glomerular thickening plus segmental hypercellularity, 2 = mild segmental hyalinosis (<25%), 2.5 = severe segmental hyalinosis (>50%), 3 = glomerular hyalinosis (‘blobs’ of hyaline material deposition), and 4 = diffuse glomerular sclerosis with total tuft obliteration and collapse, 50 glomeruli/mice, *n* = 6/group). **e**, **f** Plasma samples were collected and evaluated for total IgG and anti-β_2_GPI IgG using ELISA. **g**, **h** Kidney tissue lysates were prepared and evaluated for total IgG and anti-β_2_GPI IgG using ELISA. **i** Representative immunofluorescent staining of β_2_GPI (red), IgG (green), and nuclei (blue) in frozen kidney sections. White dashed boxes indicate the colocalization of IgG and β_2_GPI (scale bar, 25 μm). **j** Representative immunofluorescent staining of β_2_GPI (red), CD31 (**j**) (green) (scale bar, 25 μm), nephrin (**k**) (green) (scale bar, 10 μm), PDGFRβ (**l**) (green) (scale bar, 25 μm) and DAPI (blue) in frozen kidney sections. HHcy hyperhomocysteinemia, 2K1C 2-kidney, 1-clip, Cre creatinine, BUN blood urea nitrogen, UCAR urinary creatinine albumin ratio, GBM glomerular basement membrane, PDGFRβ platelet-derived growth factor receptor beta. All data are expressed as the means ± SEM. *n* = 5–6, *P < 0.05, **P < 0.01

HHcy is frequently present in patients with hypertension and exacerbates kidney damage,^6,7,38^ but the underlying mechanisms are largely unknown. To explore them, we established a mouse model of renal vascular hypertension using 2K1C surgery with drinking water supplemented with or without 1.8 g/L Hcy for 28 days (HHcy 2K1C or 2K1C mice, respectively) (Supplementary Fig. 1a). The levels of plasma Hcy were elevated in Hcy administration groups (Supplementary Fig. 1b). Hypertension and renin-angiotensin-aldosterone system (RAAS) activation were successfully induced in both HHcy 2K1C and 2K1C mice (Supplementary Fig. 1c–f). HHcy did not affect systolic blood pressure (SBP) (Supplementary Fig. 1c), which was consistent with previous studies.^39^ Plasma creatinine (Cre), blood urea nitrogen (BUN), and urinary microalbumin, the markers of renal function, were increased in 2K1C mice and were further elevated by HHcy (Fig. 1a–c). Morphologically, the kidneys on the clamped side atrophied, while the contralateral kidney showed compensatory hypertrophy (Supplementary Fig. 1g). Compared with the sham group, the contralateral kidneys of 2K1C mice showed significant glomerulosclerosis, such as fixation of partial glomerular capillaries, deposition of collagen in the renal capsule cavity, thickening of glomerular basement membrane and expansion of mesangial matrix (Fig. 1d). HHcy also induced glomerulosclerosis and further aggravated the pathological changes in 2K1C mice (Fig. 1d). Cysteine β-synthase (CBS) and cysteine γ-lyase (CSE) catalyze Hcy catabolism through the transsulfuration pathway, and inadequate systhesis of these two enzymes is often present in renal diseases.^40^ We observed that 2K1C induced downregulation of CBS but not CSE expression (Supplementary Fig. 1h, i). These results indicate that we have successfully established a HHcy 2K1C mouse model in which HHcy aggravates hypertensive renal injury.

We previously reported the pathogenic roles of Hcy-induced B-cell activation and antibody production, especially pathological anti-β_2_GPI IgG, in atherosclerosis and abdominal aortic aneurysm in humans and mice.^16,18^ In the present study, HHcy treatment significantly increased the levels of total IgG and anti-β_2_GPI IgG in plasma and kidney homogenates of sham and 2K1C mice (Fig. 1e–h). Immunofluorescence staining of kidney tissue sections showed significant colocalization of IgG and β_2_GPI in HHcy 2K1C mice, but not in 2K1C mice (Fig. 1i). β_2_GPI co-localized with CD31-positive glomerular endothelial cells (GECs) (Fig. 1j) but less with PDGFRβ-labeled glomerular mesangial cells (Supplementary Fig. 1j) and nephrin-labeled podocytes (Supplementary Fig. 1k), indicating that anti-β_2_GPI IgG deposited in GECs and possibly participated in HHcy-induced glomerular damage. To explore the roles of B cells and their anti-β_2_GPI IgG in this process, HHcy 2K1C mice were injected with rituximab (RTX, a CD20 mAbs, 75 μg/20 g body weights every other day) for 4 weeks to deplete B cells (Supplementary Fig. 2a). Flow cytometry showed that RTX significantly reduced the percentages of CD19^+^ B cells in the spleen and kidneys of HHcy 2K1C mice (Supplementary Fig. 2b, c), and that total IgG and anti-β_2_GPI IgG were also significantly reduced in plasma and kidneys (Fig. 1e–h). Plasma Cre, BUN, and urinary microalbumin levels were significantly decreased after RTX treatment (Fig. 1a–c), while glomerular basement membrane thickening and mesangial expansion were also alleviated (Fig. 1d), indicating effective recovery of renal function. RTX significantly downregulated the levels of renal inflammatory cytokines IL-1β, IL-6, and TNF-α, but had no significant effect on systemic inflammatory response (Supplementary Fig. 2d–f). Taken together, B cell-derived antibodies, especially pathogenic anti-β_2_GPI IgG, mediate HHcy-exacerbated glomerular damage in hypertensive mice.

### HHcy induces a remodeling of phospholipid composition and fatty acid accumulation in kidneys of hypertensive mice by B cell-derived antibodies

**Fig. 2:**
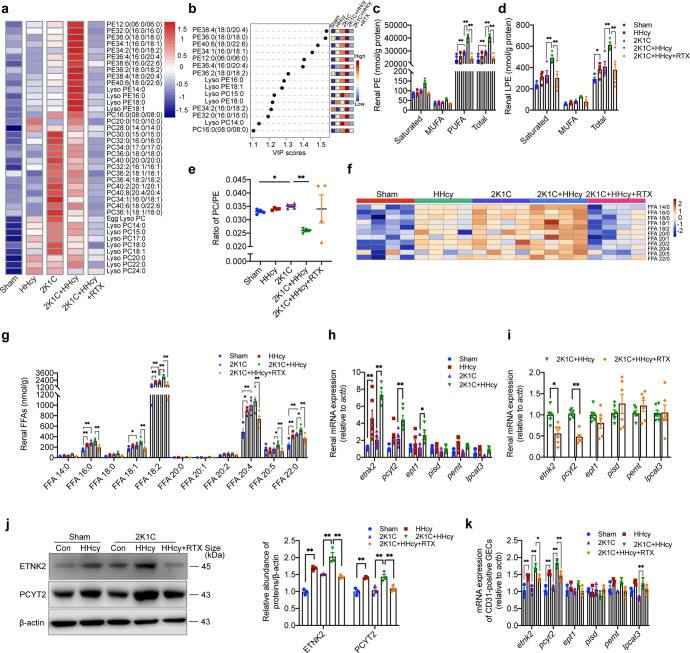
HHcy induces a remodeling of phospholipid composition and fatty acid accumulation in kidneys of hypertensive mice by B cell-derived antibodies. **a**–**g** HPLC-MS/MS analysis of lipid metabolites and free fatty acids (FFAs) in kidney tissues from sham or 2K1C mice with or without HHcy (1.8 g/L) in drinking water for 4 weeks. Rituximab was administered to HHcy 2K1C mice at the start of modeling (i.p. 75 μg/20 g body weights every other day for 4 weeks). *n* = 4. **a** Heatmap illustrating the phospholipid metabolic profiles in kidney tissues. **b** VIP scatter plot identified by PCA showing the top 15 lipid metabolites in the different groups. **c**, **d** Phospholipids were classified according to the number of double bonds in the fatty acid acyl tails and expressed as saturated, MUFA, PUFA, and total, and different species levels of PE (**c**) and LPE (**d**) in kidney tissues were analyzed using HPLC-MS/MS. **e** The ratios of total PC/PE were calculated in each group. **f** Heatmap illustrating the free fatty acids in kidney tissues. **g** Different levels of free fatty acids in kidney tissues are shown in the histogram. **h**, **i** Quantitative PCR analysis of PE and PC synthesis enzymes in the kidney (PE synthesis: etnk2, pcyt2, ept1; PC synthesis: pemt, lpcat3). *n* = 6. **j** Western blot analysis of ETNK2 and PCYT2 protein expression and quantification. β-actin was used as an internal control. *n* = 3. **k** Quantitative PCR analysis of PE and PC synthesis enzymes in the renal CD31-positive GECs from frozen section by laser capture microdissection. *n* = 3. VIP variable importance in projection, PCA principal component analysis, MUFA monounsaturated fatty acid, PUFA polyunsaturated fatty acid, etnk2 ethanolamine kinase 2, pcyt2 phosphate cytidylyltransferase 2, ept1 ethanolaminephosphotransferase 1, pemt phosphatidylethanolamine N-methyltransferase, lpcat3 lysophosphatidylcholine acyltransferase 3. All data are expressed as the means ± SEM. *P < 0.05, **P < 0.01

In view of the close relationship between lipid metabolism and HHcy-aggravated hypertensive renal injury,^33,34,36^ we used lipidomics to evaluate and identify the lipid metabolic changes. The heatmap showed a significant accumulation of 16:0, 18:0, 16:0/18:1, 16:0/18:2, 16:0/20:4, 16:0/22:6, 18:0/18:2, 18:0/20:4, 18:0/22:6 phosphatidylethanolamine (PE) and a mild decrease in phosphatidylcholine (PC) in the kidneys of HHcy 2K1C mice compared with 2K1C mice (Fig. 2a). The increase in PE-PUFAs was the most prominent, and 18:0/20:4 PE had higher variable importance in projection (VIP) scores (Fig. 2b). We further analyzed the concentrations of different phospholipid species classified according to the number of double bonds in the fatty acid acyl tail, which were showed as saturated, MUFA (monounsaturated fatty acid), PUFA, and total respectively (Fig. 2c, d). The PE-PUFA level was markedly elevated in HHcy 2K1C mice vs. 2K1C mice (Fig. 2c). However, there was no significant difference in the levels of PE-MUFAs or LPE (lysophosphatidylethanolamine)-MUFAs among these groups (Fig. 2c, d). HHcy significantly decreased the PC/PE ratio in 2K1C mice (Fig. 2e). Free fatty acids, which are substrates for phospholipid synthesis, accumulated significantly in the kidneys of HHcy mice or 2K1C mice, and 18:2 FFAs, 20:5 FFAs, and 22:0 FFAs were further elevated in HHcy 2K1C mice compared to 2K1C mice (Fig. 2f, g). PE is mainly synthesized via the Kennedy pathway (de novo pathway), and a small fraction of PE is converted from other components, such as phosphatidylserine (PS).^41^ The mRNA levels of PE synthases ethanolamine kinase 2 (*etnk2*), ethanolamine-phosphate cytidylyltransferase 2 (*pcyt2*), and ethanolamine phosphotransferase 1 (*ept1*) in the kidneys of 2K1C mice were significantly upregulated by HHcy, but the levels of phosphatidylserine decarboxylase (*pisd*) were not significantly altered among these groups (Fig. 2h). The protein expressions of etnk2 and pcyt2 were consistent with mRNA changes (Fig. 2j). Given that anti-β_2_GPI IgG deposited in GECs, we further isolated CD31-positive GECs using laser capture microdissection in frozen sections of kidney tissue to investigate their lipid synthesis gene expression induced by B-cell-derived anti-β_2_GPI IgG. The results showed that HHcy induced the upregulation of etnk2, pcyt2 and lpcat3 mRNA levels in CD31-positive glomerular endothelial cells from HHcy 2K1C mice (Fig. 2k). These results may explain the accumulation of PE-PUFA in kidney and GECs in vivo.

To further investigate whether HHcy-activated B cell-derived antibodies are involved in these changes, we evaluated the effect of RTX on HHcy-induced phospholipid remodeling. Administration of RTX in HHcy 2K1C mice resulted in a robust decrease in PE-PUFA and FFAs accumulations (Fig. 2a–g). Consistently, RTX-treated HHcy 2K1C mice showed downregulation of PE *de novo* synthase etnk2 and pcyt2 expression in kidney tissue and CD31-positive GECs (Fig. 2i–k). Therefore, HHcy provides an oxidation-prone lipid environment in the kidney of hypertensive mice by inducing B-cell activation and antibodies secretion, accompanied by increased accumulation of substrate FFAs and PE-PUFA in kidney and GECs in vivo.

### B cell-derived antibodies mediate HHcy-aggravated kidney lipid peroxidation in hypertensive mice

**Fig. 3:**
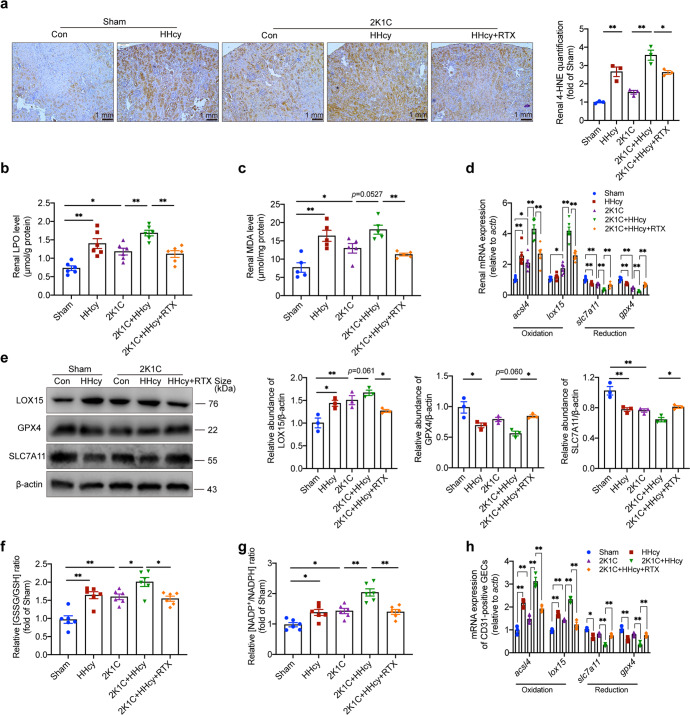
B cell-derived antibodies mediate HHcy-aggravated kidney lipid peroxidation in hypertensive mice. **a** Immunohistochemical staining of 4-HNE (brown) from kidney sections in sham or 2K1C mice with or without Hcy (1.8 g/L) in drinking water for 4 weeks, indicative of lipid peroxidation. Rituximab was administered to HHcy 2K1C mice at the start of modeling (i.p. 75 μg/20 g body weights every other day for 4 weeks). The immunohistochemical staining was calculated as the percentage of brown signals over the total area. *n* = 3. **b**, **c** Effects of 2K1C and HHcy on lipid peroxidation in the kidney were measured using ELISA for LPO (**b**) and MDA (**c**). *n* = 6. **d** Quantitative PCR analysis of redox enzyme expression in kidney tissues, including *acsl4, lox15, slc7a11* and *gpx4*. *n* = 6. **e** Western blot analysis of LOX15, GPX4 and SLC7A11 protein expression and quantification. β-actin was used as an internal control. *n* = 3. **f**, **g** The redox balance in the kidney was assayed for the ratio of GSSG/GSH and NADP^+^/NADPH using ELISA. *n* = 6. **h** Quantitative PCR analysis of redox enzyme expression in the renal CD31-positive GECs from frozen section by laser capture microdissection, including *acsl4, lox15, slc7a11* and *gpx4*. *n* = 3. LPO lipid peroxide, MDA malondialdehyde, 4-HNE 4-hydroxynonenal, lox15 lipoxygenase 15, acsl4 acyl-CoA synthetase long chain family member 4, slc7a11 cystine/glutamate transporter, gpx4 glutathione peroxidase 4, GSH glutathione, GSSG glutathione disulfide. All data are expressed as the means ± SEM. *P < 0.05, **P < 0.01

As the main component of cellular membranes, lipids have an indispensible role in maintaining the structural integrity of cells. Excessive oxidation of lipids alters the physical properties of cellular membranes and can cause covalent modification of proteins and nucleic acids.^42^ Lipid peroxidation preferentially occurs in PUFAs—long-chain fatty acids with more than one double bond.^27,42^ Indeed, immunohistochemical staining of 4-HNE in the kidneys, which is a marker of lipid peroxidation, showed a marked increase in HHcy and HHcy 2K1C mice (Fig. 3a). HHcy and 2K1C treatment significantly increased renal LPO and MDA, the products of lipid peroxidation, and their levels were higher in HHcy 2K1C mice compared to 2K1C mice (Fig. 3b, c). The ACSL4 (long-chain fatty acid CoA ligase 4) mRNA level and LOX15 expression, catalyzing PUFAs activation and lipid peroxidation, were elevated in kidney tissues of HHcy 2K1C mice compared to 2K1C mice (Fig. 3d, e). The mRNA and protein levels of SLC7A11, the catalytic subunit of cystine/glutamate antiporter System Xc^−^, were downregulated in kidney tissues of 2K1C mice compared to the sham mice and further reduced by HHcy treatment (Fig. 3d, e). HHcy significantly decreased the gene expression of GPX4 in HHcy 2K1C mice compared to 2K1C mice, with a down-regulation trend in protein levels (Fig. 3d, e). The ratios of the main cellular redox couples, GSSG/GSH and NADP^+^/NADPH,^43^ were significantly increased in 2K1C mice, which were further aggravated by HHcy (Fig. 3f, g). In addition, HHcy also induced upregulation of *acsl4* and *lox15* mRNA levels and downregulation of *slc7a11* and *gpx4* mRNA levels in CD31-positive GECs in the HHcy and 2K1C HHcy mice (Fig. 3h). These results suggest that HHcy induces the perturbation of redox equilibrium and aggravates the renal lipid peroxidation of hypertensive mice.

The effect of HHcy-activated B cell-derived antibodies on lipid peroxidation was validated by depletion of B cells and showed that renal lipid peroxidation was diminished in HHcy 2K1C mice treated with RTX (Fig. 3a–c). In addition, RTX ameliorated the redox imbalance in kidneys and GECs of HHcy 2K1C mice (Fig. 3d–h). Together, these results indicate that targeting B cells is instrumental for the inhibition of HHcy-exacerbated lipid peroxidation in the kidneys and GECs of hypertensive mice.

### Targeting B cell-derived antibodies improves HHcy-aggravated iron accumulation in hypertensive mice

**Fig. 4:**
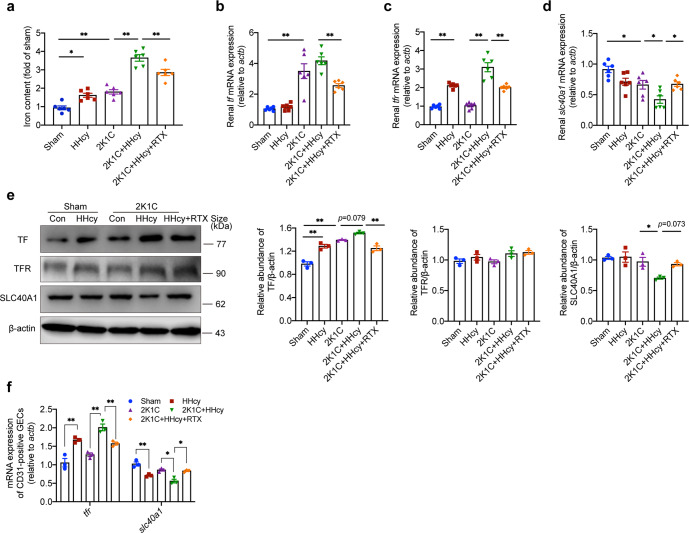
Targeting B cell-derived antibodies to improve HHcy-aggravated iron accumulation in hypertensive mice. Iron content and metabolism were analyzed in mice treated with sham or 2K1C surgery and given drinking water with or without Hcy (1.8 g/L) for 4 weeks. Rituximab was administered to HHcy 2K1C mice (i.p. 75 μg/20 g body weights every other day for 4 weeks). **a** Iron concentrations in kidney tissues were measured using ELISA. *n* = 6. **b**–**d** Quantitative PCR analysis of the genes associated with iron metabolism in kidney tissues, including *tf* (**b**), *tfr* (**c**) and *slc40a1* (**d**). *n* = 6. **e** Western blot analysis of TF, TFR, and SLC40A1 protein expression and quantification in kidney tissues. β-actin was used as an internal control. *n* = 3. **f** Quantitative PCR analysis of the genes associated with iron metabolism in the renal CD31-positive GECs from frozen section by laser capture microdissection, including *tfr* and *slc40a1*. *n* = 3. TF transferrin, TFR transferrin protein receptor, slc40a1 solute carrier family 40 member 1, HE staining hematoxylin and eosin staining, PAS staining periodic acid-Schiff staining. All data are expressed as the means ± SEM. *P < 0.05, **P < 0.01

Lipid peroxides are not only key mediators of many pathological states, including inflammation and renal degeneration, but have recently been identified as a key downstream feature of ferroptosis, an emerging form of regulated nonapoptotic cell death.^44^ Iron accumulation is another important manifestation of ferroptosis, which interferes with redox homeostasis, catalyzes ROS propagation, leading to oxidative stress and tissue damage.^45^ To explore whether ferroptosis participates in HHcy-aggravated hypertensive renal injury, we further detected the levels of iron. Indeed, ELISA showed that HHcy promoted iron accumulation in the kidneys of HHcy 2K1C mice compared to 2K1C mice, and RTX effectively alleviated this alteration (Fig. 4a). Abnormal iron accumulation due to loss of balance between influx and efflux. Transferrin (TF) delivers large amount of iron from plasma to the bone marrow for heme biosynthesis and small amount to other tissues, which is responsible for iron influx into cells.^45^ The mRNA and protein levels of TF were significantly increased in the kidneys of 2K1C mice compared to sham mice, and there was a trend of further increase in HHcy 2K1C mice (Fig. 4b, e). HHcy treatment upregulated transferrin receptor (TFR) mRNA expression in the kidneys of sham and 2K1C mice, but did not affect TFR protein expression (Fig. 4c, e). However, the mRNA and protein levels of SLC40A1, which mediates iron efflux from cells,^45^ were decreased in HHcy 2K1C mice compared to 2K1C mice (Fig. 4d, e). After RTX treatment, the alteration of TF, TFR, and SLC40A1 in mRNA and protein levels in the kidney tissues of HHcy 2K1C mice were partial ameliorated, except for TFR protein level (Fig. 4b–e). Similarly, HHcy treatment significantly increased TFR gene expression and decreased SLC40A1 gene levels in HHcy and HHcy 2K1C renal CD31-positive GECs, and RTX treatment effectively attenuated these changes (Fig. 4f). Taken together, these results suggest that HHcy promotes renal and GEC iron deposition and might trigger ferroptosis during renal injury in 2K1C mice via B-cell-derived antibodies.

### Ferroptosis mediates GECs dysfunction induced by anti-β_2_GPI antibody derived from Hcy-activated B cells in vitro

**Fig. 5:**
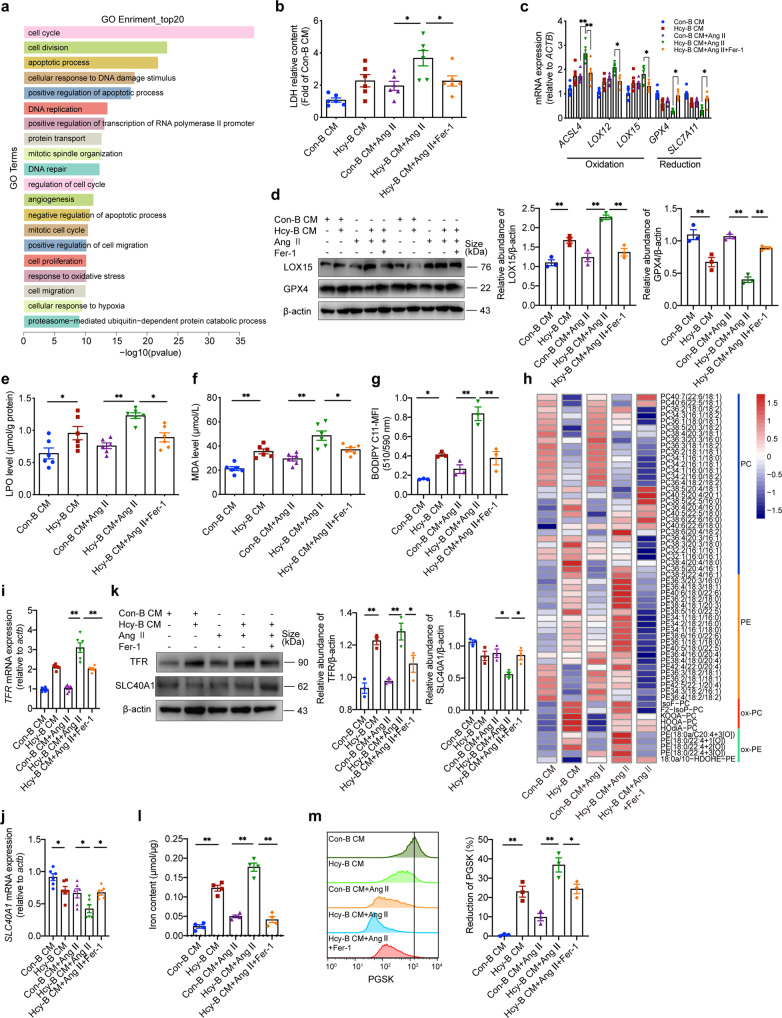
Ferroptosis mediates GECs dysfunction induced by anti-β2GPI antibody derived from Hcy-activated B cells in vitro. We evaluated the bioactive effects of Hcy-activated B cell-derived antibodies, particularly anti-β_2_GPI, on GECs. Ferrostatin-1 (Fer-1, 5 μM) was administered to Hcy-B CM + Ang II GECs to inhibit ferroptosis. **a** Cellular RNA was sequenced by RNA‐Seq, and the top 20 GO enrichment results between the differentially expressed genes in con-B CM + Ang II and Hcy-B CM + Ang II treated GECs were shown. **b** LDH release into GEC culture medium was measured using an ELISA kit. *n* = 6. **c** Quantitative PCR analysis of redox enzyme expression in GECs, including ACSL4, LOX12, LOX15, GPX4, and SLC7A11. *n* = 6. **d** Western blot analysis of LOX15 and GPX4 protein expression and quantification. β-actin was used as an internal control. *n* = 3. **e**, **f** The intracellular LPO (**e**) and MDA (f) levels in GECs were measured using ELISA. *n* = 6. **g** For flow cytometry analysis, GECs were treated with or without Ang II (1 μM) and with or without Hcy (100 μM)-activated B cell culture supernatant for 24 h, and 5 μM BODIPY-C11 dye was added during the last hour and resuspended in culture medium. The cells were washed twice with ice-cold PBS, stained with 7-AAD for 5 min, trypsinized and filtered into single-cell suspensions. Flow cytometry analysis was performed using a PE-Texas Red filter for reduced BODIPY-C11 and an FITC filter for oxidized BODIPY-C11. **h** HPLC-MS/MS analysis of phospholipids and oxidized phospholipids in GECs from each group. Heatmap illustrating the phospholipid metabolic profiles in GECs. *n* = 4. **i**, **j** Quantitative PCR analysis of TFR (**i**) and SLC40A1 (**j**) associated with iron metabolism in GECs. *n* = 6. **k** Western blot analysis of TFR and SLC40A1 protein expression and quantification in kidney tissues. β-actin was used as an internal control. *n* = 3. **l**, **m** Intracellular iron concentrations in GECs were measured using ELISA (**l**, *n* = 4) and Phen Green SK (PGSK) staining was assessed using flow cytometry (**m**, *n* = 3). Higher Fe^2+^ concentrations are indicated by weaker PGSK fluorescence intensity. The reductions in PGSK fluorescence intensity were calculated. NHIgG and aPL treatment of GECs. All data are expressed as the means ± SEM. *P < 0.05, **P < 0.01

Considering the deposition of anti-β_2_GPI IgG on CD31-positive GECs, we examined the effects of Hcy-induced B cell-derived-anti-β_2_GPI IgG on GECs in vitro. We have demonstrated that the culture medium of B cells treated with 100 μM Hcy for 72 h, which contains high level anti-β_2_GPI antibody, and its purified IgG both induced a shift in macrophage phenotypes towards M1 polarity and evoked vascular inflammation.^18^ GECs were treated with con-B CM or Hcy-B CM (culture medium of B cells treated with or without 100 μM Hcy for 72 h) and with or without Ang II for 24 h to simulate the in vivo environment of renal hypertension and HHcy (Supplementary Fig. 3a). We used 1 μM Ang II as the stimulation concentration because GEC viability began to decrease in the CCK-8 assay at this concentration (Supplementary Fig. 3b). The concentration of Hcy in Hcy-B CM was about 0.4167 ± 0.2638 μM (Supplementary Fig. 3c), which had no effect on the viability of GECs (Supplementary Fig. 3d), so the potential effect of residual Hcy was excluded. The results from RNA-sequencing (RNA-seq) showed that the top 20 GO enrichment results between the differentially expressed genes in con-B CM + Ang II and Hcy-B CM + Ang II treated GECs included cell cycle, apoptotic processes, oxidative stress and hypoxia, indicating that multiple cellular damage pathways were activated in Hcy-B CM + Ang II treated GECs (Fig. 5a). Indeed, the LDH level in the Hcy-B CM + Ang II group was significantly higher than the Ang II alone group (Fig. 5b), and ferroptosis inhibitor ferrostatin-1 (Fer-1, 5 μM) rescued this change (Fig. 5b). In addition, Hcy-B CM increased the percentage of Annexin V^+^ GECs induced by Ang II, whereas the ferroptosis inhibitors Fer-1 and liproxstatin-1 (Lip-1, 200 nM) inhibited this effect (Supplementary Fig. 3e). We assessed the redox state of GECs under different stimulations. The mRNA levels of the lipid peroxidation-related enzymes ACSL4, LOX12, and LOX15 were increased, and the mRNA expression levels of the GPX4 and SLC7A11, antioxidant enzyme and transporter, were decreased in the Hcy-B CM + Ang II group compared to the Ang II alone group (Fig. 5c). The changes in protein expression of GPX4 and LOX15 paralleled the changes in mRNA expression, suggesting the increased oxidative and diminished antioxidant capacities (Fig. 5d). Besides, the levels of LPO and MDA in the Hcy-B CM + Ang II group were markedly increased compared to the Ang II alone group (Fig. 5e, f). Hcy-B CM treatment aggravated the accumulation of lipid peroxides in the Hcy-B CM + Ang II group vs. the Ang II alone group, as revealed by BODIPY-C11 staining in GECs (Fig. 5g and Supplementary Fig. 3f). The oxidative phospholipidomic assays provided further direct evidences of increased oxygenated phospholipids, including oxPE and oxPC in the Hcy-B CM + Ang II group of GECs (Fig. 5h).

Iron metabolism was also assessed in GECs. The mRNA and protein expression of transferrin receptor TFR were increased, and the expression of iron efflux transporter SLC40A1 was decreased in the Hcy-B CM + Ang II group compared to the Ang II alone group (Fig. 5i–k). ELISA assay showed that Hcy-B CM increased the intracellular Fe^2+^ concentration in the Hcy-B CM + Ang II group of GECs (Fig. 5l). PGSK probe staining further validated these changes in the Fe^2+^ concentration using flow cytometry analysis and immunofluorescence staining, which showed a higher Fe^2+^ concentrations indicated by weaker fluorescence intensity (Fig. 5m and Supplementary Fig. 3g). Fer-1 treatment (5 μM, 24 h) reversed the expression of the antioxidants GPX4 and SLC7A11 and the oxidants ACSL4, LOX12, and LOX15 (Fig. 5c, d). ELISA and BODIPY-C11 staining showed that Fer-1 treatment reduced lipid peroxidation in the Hcy-B CM + Ang II group of GECs (Fig. 5e–g and Supplementary Fig. 3f), which was directly demonstrated by oxidative phospholipidomics (Fig. 5h). Fer-1 treatment also ameliorated the upregulated expression of TFR and downregulated expression of SLC40A1 (Fig. 5i–k), which contributed to the iron reduction in Fer-1-treated GECs (Fig. 5l, m and Supplementary Fig. 3g). Taken together, these results suggest that ferroptosis mediates the dysfunction of GECs caused by Hcy-activated B cell-derived antibodies, primarily pathogenic anti-β_2_GPI IgG, with iron-dependent oxidative phospholipid induction and lipid peroxidation.

### aPL induces ferroptosis in GECs in vitro

**Fig. 6:**
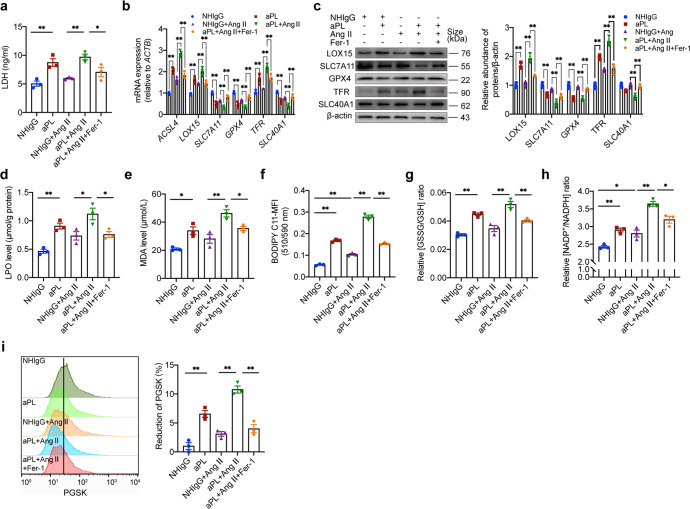
aPL induce ferroptosis in GECs in vitro. Evaluation of purified antiphospholipid antibodies (aPL, 100 μg/ml) and control IgG (NHIgG, 100 μg/ml) on GECs. **a** LDH release was measured using an ELISA kit. *n* = 3. **b**, **c** Quantitative PCR and western blot analysis of redox and iron metabolism enzymes expression in GECs, including ACSL4, LOX15, GPX4, SLC7A11, TFR, and SLC40A1. **d**, **e** The intracellular LPO (**d**) and MDA (**e**) levels in GECs were measured using ELISA. *n* = 3. **f** Detection of lipid peroxides by BODOPY-C11. *n* = 3. **g**, **h** Intracellular GSSG/GSH (**g**) and NADP^+^/NADPH (**h**) ratios in GECs were measured using ELISA. *n* = 3. **i** Intracellular iron concentrations in GECs were measured using PGSK staining. *n* = 3. LDH lactate dehydrogenase. All data are expressed as the means ± SEM. *P < 0.05, **P < 0.01

To further illustrate the function of B-cell-derived anti-β_2_GPI IgG, we used purified antiphospholipid antibodies (aPL, 100 μg/ml) and control IgG (NHIgG, 100 μg/ml) to treat GECs for 24 h. aPL significantly induces LDH release from GECs, and Fer-1 (5 μM) effectively ameliorates GECs impairment (Fig. 6a). Further analysis of redox-related enzyme expression showed that aPL significantly upregulated gene and protein expression of ACSL4 and LOX15, and decreased the expression of SLC7A11 and GPX4 (Fig. 6b, c). In this oxidative environment, GECs showed lipid peroxide accumulation induced by aPL (Fig. 6d–f). Fer-1 treatment effectively improved the abnormal expression of redox-related enzymes and reduced lipid peroxide levels in GECs (Fig. 6b–f). aPL-induced imbalance in GSSG/GSH and NADP^+^/NADPH ratios was also effectively alleviated by Fer-1 treatment (Fig. 6g, h). Furthermore, aPL-induced upregulation of TFR expression and downregulation of SLC40A1 in GECs, and intracellular Fe^2+^ levels were significantly increased in the presence of aPL (Fig. 6b–c, i). However, Fer-1 treatment significantly ameliorated these changes (Fig. 6b–c, i). Thus, these results provide evidence that antiphospholipid antibodies cause ferroptosis in GECs.

### Ferroptosis mediates HHcy-exacerbated kidney injury in vivo

**Fig. 7:**
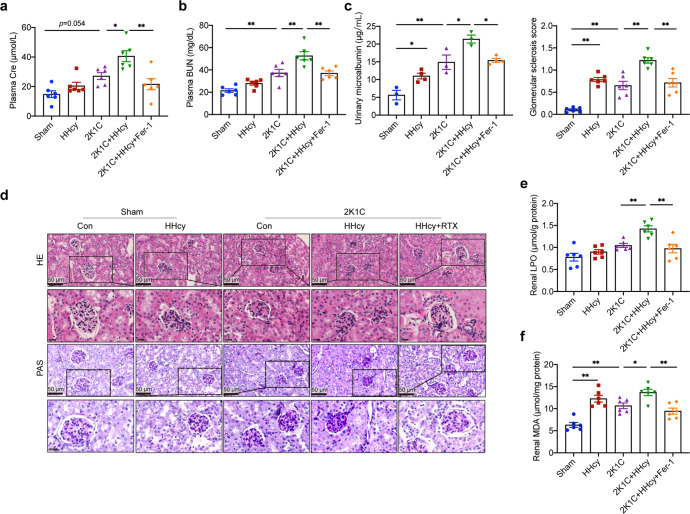
Ferroptosis mediates HHcy-exacerbated kidney injury. Fer-1, a specific ferroptosis inhibitor, was administered to HHcy 2K1C mice at the start of modeling (1 mg/kg/day, i.p. 4 weeks) to evaluate the effect of ferroptosis on kidney damage. **a**–**c** Plasma Cre (**a**), BUN (**b**), and urinary microalbumin (**c**) were assayed to evaluate renal function using ELISA. *n* = 6. **d** Representative HE staining (top) and PAS staining (bottom) (scale bar, 50 μm) in kidney paraffin sections. Quantification of the glomerular matrix index was performed for each group (50 glomeruli/mice, *n* = 6/group). **e**, **f** Kidney tissue lysates were prepared and evaluated for LPO (**e**) and MDA (**f**) using ELISA. *n* = 6. All data are expressed as the means ± SEM. *P < 0.05, **P < 0.01

Lipid peroxidation and iron accumulation are characteristic manifestations of ferroptosis.^44^ We further determined whether ferroptosis is involved in the pathogenesis of hypertensive renal injury exacerbated by HHcy by pharmacological methods. HHcy 2K1C mice were continuously injected with the ferroptosis inhibitor ferrostatin-1 (Fer-1, 1 mg/kg/day, i.p.) for 4 weeks (Supplementary Fig. 4). ELISA showed a significant reduction in plasma Cre, BUN and urinary microalbumin after Fer-1 treatment in HHcy 2K1C mice (Fig. 7a–c). H&E and PAS staining showed that Fer-1 treatment significantly improved the exacerbated glomerulosclerosis in HHcy 2K1C mice (Fig. 7d). Fer-1 also inhibited the renal increased LPO and MDA levels in HHcy 2K1C mice (Fig. 7e, f). These results indicate that ferroptosis mediates the pathogenesis of HHcy-exacerbated hypertensive renal injury.

## Discussion

**Fig. 8:**
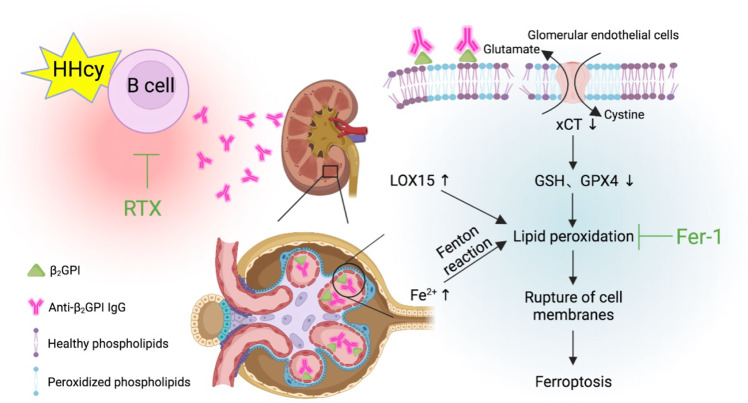
Schematic diagram. HHcy aggravates hypertensive kidney damage by activating B lymphocytes to secrete pathological anti-β_2_GPI IgG, which promotes lipid peroxidation and ferroptosis in hypertensive glomerular endothelial cells

CKD has become a leading cause of morbidity and mortality worldwide in the last few decades.^11,46^ A previous epidemiological study reveals that HHcy predicts reduced renal function and the incidence of CKD in hypertensive patients.^47^ Immune-associated glomerulonephritis is a major cause of CKD, and its pathogenesis is based on the interaction between bone-marrow-derived immune cells and cells intrinsic to the kidney.^48^ Notably, B lymphocytes and their antibodies are key players in immune activation.^48,49^ In the present study, we found, from an immunological perspective, that HHcy activated B cells and promoted antibody production and secretion, especially the pathogenic anti-phospholipid binding protein β_2_GPI IgG, which deposited in glomerular endothelial cells to induce ferroptosis (Fig. 8). Improvements in HHcy-aggravated renal dysfunction via B cell depletion using RTX further confirmed that B cells and anti-β_2_GPI IgG-related immune mechanisms were the main pathways in HHcy-exacerbated hypertensive kidney injury.

Anti-β_2_GPI antibodies are a class of antiphospholipid antibodies (aPLs), which are the biomarker in the serum of patients with cardiovascular disease, mediating the development of autoimmune disease antiphospholipid syndrome (APS).^50^ As the major membrane-bound antigenic protein of aPL, β_2_GPI forms a specific immune complex with serum anti-β_2_GPI antibodies, which is closely related to systemic lupus erythematosus (SLE) and atherosclerosis through TLR signaling.^51,52^ TLRs are a major class of germ-line encoded receptors that activate the B-cell-mediated pathological process in autoimmune diseases. In our previous work, we have shown that anti-β_2_GPI antibodies produced by Hcy-activated B cells polarizes macrophages to M1 through TLR4 signaling pathway and contributes to AAA, and β_2_GPI-expressing endothelial cells may also be involved.^18^ This is consistent with the reports that anti-β_2_GPI antibodies mediate proinflammatory phenotype of endothelial cells and monocytes.^51,53^ The cationic phospholipid-binding site (located in the fifth domain of the molecule) and anionic structures, such as heparin sulfate, on the cell membrane, interact electrostatically to anchor β_2_GPI to endothelial cell membranes I or as a ligand for annexin A2.^54^ Anti-β_2_GPI antibody binding results in clustering of β_2_GPI with its potential receptors, such as Toll-like receptor (TLR) 4, annexin A2, and apolipoprotein E receptor 2, and it also triggers cell signal transduction, activating either p38 mitogen-activated protein kinase (MAPK) or nuclear factor κB (NFκB) or both.^54,55^ In our study, we demonstrated that ferroptosis is an important novel mechanism by which anti-β_2_GPI/β_2_GPI immune complex disrupts GECs function. Although circulating aPLs and endothelial dysfunction are necessary “first hits” for cardiovascular events in APS, an inflammatory “second hit” to upregulate the expression of β_2_GP1 receptors on the endothelial cell surface is needed.^54^ The “second hit” may be consistent with the elevated blood pressure induced by 2K1C, and it may account for the higher susceptibility of 2K1C mice to suffer an HHcy-induced decline in renal function. This hypothesis is supported by a recent report that the strength of the relationship between plasma Hcy levels and cardiovascular events is greater in patients with hypertension than patients without hypertension.^56^

Immune system, as the main defense mechanism of the body, can be induced rapid adaptive response of metabolic remodeling in immune cells by immunogenic stimulation, and this process is called immune metabolism.^57^ We have demonstrated that glucose metabolism and accumulation of phospholipids and fatty acids mediates HHcy-induced B cell activation and Ab secretion, including pathogenic anti-β_2_GPI antibodies.^17,18^ Glucose-dependent *de novo* lipogenesis supports proliferation and expansion of the cellular endoplasmic reticulum and Golgi endomembrane network, which favors B-cell Ab production and secretion.^58^ However, the activation of liver X receptor to increase cholesterol and fatty acid excretion and activation of fatty acid oxidation by the peroxisome proliferator activated receptor alpha (PPARα) agonist fenofibrate both effectively inhibit Hcy-induced B cell IgG and anti-β_2_GPI IgG secretion,^18,59^ indicating the importance of B cell lipid accumulation and metabolism for anti-β_2_GPI IgG production and secretion. More importantly, when endothelial cells or monocytes/macrophages are subjected to stress, membrane phospholipids are perturbed to expose β_2_GPI binding sites and recruit circulating anti-β_2_GPI antibodies to form immune complexes that mediate cell signal transduction via the potential receptor TLR4.^60^ In the present study, we found that the anti-β_2_GPI IgG binded to the glomerular endothelial cell antigen β_2_GPI, and depletion of B cells by RTX confirmed that this immune complex was involved in hypertensive renal injury exacerbated by HHcy. Furthermore, we demonstrated that HHcy-activated B cell-derived antibodies mediate macrophage polarization toward inflammatory M1 via F(ab′)2, suggesting an antigen-dependent mode of action.^18^ RNA-seq results showed that blocking TLR4 (TAK-242 pretreatment, 10 nM, 30 min) effectively inhibited fatty acid transport and synthesis, phospholipid synthesis, and iron transport into cells, providing strong evidence that TLR4 is a potential receptor for anti-β_2_GPI antigen-antibody complexes (data not shown).

As a major risk factor for chronic kidney disease, disorders of lipid metabolism induce structural damage and dysfunction of biological membranes and reactive phospholipid products that act as signaling molecules induce oxidative stress and inflammation.^61–64^ The present study reported lipid remodeling in the renal tissue of HHcy 2K1C mice, particularly polyunsaturated PE accumulation and a decreased PC/PE ratio compared to 2K1C mice. The key enzymes that catalyze PE synthesis, etnk1/2, pcyt2 and ept1, were upregulated in HHcy 2K1C mice. This indicates an increase in *de novo* PE synthesis, especially polyunsaturated PE. In HHcy 2K1C mice, ACSL4, which favors the substrate arachidonate, was also increased. ACSL4 catalyzes the conversion of long-chain fatty acids to their active form, acyl-CoA, for the synthesis of cellular lipids.^65^ These results are consistent with our results of significant increases in renal 16/0–20/4 PE and 18/0–20/4 PE (Fig. 2a, b). Knockdown of ACSL4 reduces the cellular PUFA phospholipid content.^66^ Abundant FFAs provide sufficient substrates for PE-PUFA synthesis (Fig. 2f, g). The increase in PE-PUFA enhances the sensitivity of PE to oxidative stress attack.^27^ LPC levels were also slightly increased in the kidneys of HHcy mice compared with sham mice. The generation of LPC is dependent on phospholipase A2 (PLA2) to hydrolyze the sn-2 position of PC. We have demonstrated that LPC had a pro-inflammatory effect in adipocytes.^36^ This effect may be one of the mechanisms for renal inflammation in renal tissue. Renal lipid metabolism disorders and FFAs accumulation were effectively ameliorated in HHcy 2K1C mice when RTX treatment was administered. However, the regulatory mechanism of the decrease in the PC/PE ratio caused by HHcy-activated B cell-derived antibodies requires further investigation.

The close association of PE-PUFA with ferroptosis indicated by KEGG signaling pathway analysis attracted our attention. Ferroptosis is a regulated form of cell death that occurs when PL-PUFAs are oxidized in an iron-dependent manner,^23,25^ and it is gaining attention in many kidney diseases, such as ischemic kidney injury and renal cell carcinoma.^67–69^ As substrates for lipid peroxidation, PL-PUFAs are susceptible to attack by oxidants, such as free radicals. The presence of aPL provides an oxidative stress environment.^70,71^ Initial lipid hydroperoxides (LOOHs), following reactive aldehydes, MDA, and 4-HNE are all byproducts of lipid peroxidation. We found that Hcy-treated B cell culture supernatant containing abundant anti-β_2_GPI antibodies contributed to lipid peroxidation in GECs in vitro. The oxidative phospholipidomics results further confirmed with a significant increase in oxidized PC and oxidized PE, such as IsoF-PC, F2-IsoP-PC, PE(18:0a/C20:4 + 3[O]), PE(18:0/C22:4 + 1[O]) PE(18:0/C22:4 + 2[O]), and PE(18:0/C22:4 + 3[O]) (Fig. 5h). After being incorporated into membrane environments, PL-PUFAs undergo a peroxidation reaction with labile iron and iron-dependent enzymes to produce PL-PUFA-OOH, which is sufficient to damage the membrane and trigger the cell death program.^25,26^ Initiating membrane damage during ferroptosis only requires 2% oxidative damage to PUFAs.^72^ Ferroptosis is fueled by iron-dependent enzymes such as lipoxygenases and cytochrome P450 oxidoreductase (POR).^72,73^ We showed that 12-lipoxygenase (LOX12) and LOX15 expression were upregulated in vitro and in vivo. LOX15 has catalytic competence in the selective oxidation of membrane ETE-PE to ferroptosis signals, HpETE-PE.^74^ Fer-1, a well-known inhibitor of ferroptosis, exhibits anti-ferroptotic activity because it scavenges the initiating alkoxyl radicals and other rearrangement products that ferrous iron from lipid hydroperoxides produces.^75^ In addition, Fer-1 has been reported to effectively reduce the intracellular labile iron pool by forming a complex with Fe^2+^,^75^ which partially explains our observation that Fer-1 treatment reduces Fe^2+^ levels in vivo and in vitro. Further studies demonstrated that Fer-1 did not affect LOX15 alone, but it effectively inhibited HpETE-PE production by the LOX15/phosphatidylethanolamine binding protein-1 (PEBP1) complex.^76^ Fer-1 treatment in our study significantly attenuated the decline in renal function in vivo and oxidative phospholipids of GECs in vitro, which confirmed that ferroptosis mediated the anti-β_2_GPI IgG-induced peroxidation of phospholipids and renal injury.

Through the transferrin receptor 1 (TFR1), labile iron is imported and stored in ferritin. The process of ferritin degradation, known as ferritinophagy, releases labile iron and promotes the peroxidation reaction that leads to ferroptosis.^77,78^ The activity of iron-dependent enzymes is also dependent on iron. Lipid peroxidation is a consequence of iron accumulation and may be a trigger to regulate iron metabolism by affecting membrane fluidity and membrane protein homeostasis, such as related receptors and transporters, as evidenced in a recent report.^79^ Renal CD31-positive GECs obtained by laser microdissection showed upregulation of TFR and inhibition of SLC40A1, a membrane transporter that mediates iron efflux, was induced by HHcy (Fig. 4f). Similarly, we observed consistent results in Hcy-B CM-treated GECs in vitro (Fig. 5i–k). These changes together lead to iron overload in GECs.

GPX4 is one of the most important antioxidant enzymes, and it uses the cysteine-containing tripeptide glutathione (GSH) to eliminate phospholipid peroxides. The cystine/glutamate transporter system Xc^−^ exchanges intracellular glutamate for extracellular cystine for GSH synthesis.^24^ GPX4 activity and stability are directly impacted by GSH depletion, which makes cells more susceptible to ferroptosis.^80^ HHcy induced an increase in the GSSG/GSH ratio and a downregulation of GPX expression in our study due to the downregulation of system Xc^−^. Intracellular cysteine also originates from the transsulfuration pathway catalyzed by CBS, which is constantly activated in some tumor cells.^81^ We showed that reduced renal CBS expression may further accelerate GSH depletion and weaken the antioxidant capacity in hypertensive kidneys. Iron accumulation also inhibits antioxidant capacity.

In conclusion, HHcy aggravates hypertensive kidney damage by activating B lymphocytes to secrete pathological anti-β_2_GPI IgG, which promotes PE-PUFA, as substrates and iron-dependent lipid peroxidation, thus ferroptosis in hypertensive glomerular endothelial cells. This study provides therapeutic guidance for nephropathy patients with HHcy, particularly in aging patients with likely CBS deficiency. The suppression of B lymphocyte hyperactivation or ferroptosis may be an effective therapeutic strategy in HHcy with CKD patients.

## Materials and methods

### Reagents and antibodies

DL-homocysteine (Hcy, H4628) was purchased from Sigma–Aldrich (St. Louis, MO, USA). Rituximab (RTX, 10 mg/ml), a monoclonal anti-human CD20 antibody, was purchased from Roche (Basel, Switzerland). Ferrostatin-1 (Fer-1, S7243) and liproxstatin-1 (lip-1, S7699) were purchased from Selleckchem (Houston, TX, USA). The following antibodies were used in this work. Anti-β_2_GPI (bs-1570R) was purchased from Bioss Inc. (Beijing, China). Anti-CBS (cystathionine β-synthase, 14787-1-AP) and anti-CSE (cystathionine γ-lyase, 12217-1-AP) were purchased from Proteintech (Rosemont, IL, USA). Anti-CD31 (sc-18916), anti-nephrin (sc-377246) and anti-PDGFRβ (platelet-derived growth factor receptor beta, sc-374573) were purchased from Santa Cruz Biotech (CA, USA). Anti-4-HNE (4-hydroxynonenal, MAB3249) was purchased from R&D Systems (MN, USA). Anti-xCT (catalytic subunit of cystine/glutamate antiporter System Xc-, also called SLC7A11, ab37185), anti-LOX15 (Lipoxygenase 15, ab23691), anti-GPX4 (glutathione peroxidase 4, ab125066), and anti-TFR (transferrin receptor, ab214039) were purchased from Abcam (Cambridge, MA, USA). Anti-TF (transferrin, A1448), anti-SLC40A1 (A14884), anti-β-actin (AC038), HRP-conjugated goat anti-rabbit (AS014), and HRP-conjugated goat anti-mouse (AS003) were purchased from ABclonal (Wuhan, China).

### Animal models

Male C57BL/6J mice (8 weeks old) received standard or DL-Hcy (1.8 g/L)-containing drinking water for 4 weeks to establish the HHcy model as previously described.^18^ A 0.12-mm silver clip was placed on the left renal artery to operate 2-kidney, 1-clip (2K1C). The method of the 2K1C model was modified from a previous report.^82^ Blood pressures of these mice were measured before surgery and post-operatively twice weekly using a tail-cuff blood pressure (BP) measurement system (Kent Scientific Corporation). Kidney tissue was collected 4 weeks after surgery. Plasma renin, Ang II and aldosterone levels were determined to assess RAAS activity using enzyme-linked immunosorbent assay kits (Dogesce, Beijing, China). All animal experiments were carried out in accordance with the Institute of Laboratory Animal Resources and with the approval of Peking University’s Animal Care and Use Committee.

### Renal function detection

Plasma creatinine levels were determined using a colorimetric assay kit (C011-1, Jiancheng, Nanjing, China). Plasma urea concentrations were analyzed using the QuantiChromTM Urea Assay Kit (BioAssay Systems DIUR-500). Random urine was collected, and the levels of microalbuminuria in urine were determined using a mouse microalbuminuria ELISA kit (EIA06046, Xinqidi, Wuhan, China).

### Histological analysis

Paraffin embedding and sectioning were performed on kidney specimens. Ten parts each segment/interval (5 μm) were collected at intervals of 50 μm. Periodic acid Schiff (PAS) and hematoxylin and eosin (H&E) stains were used to identify the architecture of the kidneys. Glomerulosclerosis was examined to gauge the extent of renal injury. Each kidney slide had fifty glomeruli examined to determine its glomerulosclerosis score, which ranged from 0 for normal glomeruli to, 1 for thickening of the GBM, 1.5 for glomerular thickening combined with segmental hypercellularity, 2 for mild segmental hyalinosis (<25%), 2.5 for severe segmental hyalinosis (>50%), 3 for glomerular hyalinosis (or “blobs” of hyaline material deposition) to 4 for diffuse glomerular sclerosis with total tuft obliteration and collapse.^83^ Six animals per experimental group were examined, and each animal had fifty glomeruli counted. The average of these ratings for each experimental group is used to present the data.

### Measurement of antibody levels

Utilizing mouse ELISA kits, total IgG and anti-β_2_GPI IgG concentrations in plasma and kidney tissue homogenate supernatants were examined (Bethyl Laboratories, Montgomery, TX, USA).

### Laser capture microdissection of frozen tissue sections

7 μm-thick frozen sections were attached to microscope slides that had been precoated with polyethylene naphthalate for immunofluorescence labeling. Using a Lecia LMD6000 system (Leica, Wetzlar, Germany) in a laminar flow biosafety cabinet, laser microdissection and laser pressure catapulting were carried out to capture CD31-positive glomerular endothelial cells in the tissue slices under fluorescence microscopy. Pure cells or tissues were removed from the slides using a 337 nm pulsed UV laser, collected in a sample tube with trizol, and then were treated to RNA extraction and reversal.

### Cell lines

Purifying magnetic microbeads with anti-CD19 antibodies (Miltenyi Biotec, Bergisch Gladbach, Germany) were used to separate splenic B cells. The RPMI-1640 medium supplemented with 10% fetal bovine serum (FBS, Gibco, Grand Island, NY), and 0.1 mg/mL lipopolysaccharide (LPS, Sigma Aldrich Corporation, St. Louis, MO, USA) was used to culture the purified B cells. Prior to the following measurements, the B cells were either treated with or without 100 μM Hcy.^18^ Human renal glomerular endothelial cells (HGECs) (ScienCell, 4000) were cultured in endothelial cell medium (ScienCell, 1001) containing 5% FBS and 1% endothelial cell growth supplement (ECGS). When cell confluency reached 70–80%, the cells were treated with or without Ang II (1 μM) and with or without a 50% volume of culture supernatant from Hcy-activated B cells for 24 h to simulate the renal hypertension environment and measure the bioactive effects of anti-β_2_GPI-derived culture supernatants of Hcy-activated B cells. Stimulation concentrations and times were determined using Cell Counting Kit-8 (CCK-8) assays (Solarbio, Beijing, China).

Purified anti-phospholipid antibodies (aPL, 100 μg/ml) and control IgG (NHIgG, 100 μg/ml) from the Department of Rheumatology, Peking University People’s Hospital were utilized to treat GECs for 24 h. Healthy non-autoimmune persons provided NHIgG. Patients with APS provided aPL.^84^

### Ferroptosis assay

For HHcy 2K1C mice, Fer-1 (1 mg/kg/day, i.p.) was used for 4 weeks. HGECs were treated with or without Fer-1 (5 μM) or liproxstatin-1 (200 nM) for 24 h in the absence of 10^−6^ M Ang II and 50% volumes of culture supernatant from Hcy-activated B cells. Cell death was detected by LDH release using the LDH Detection Kit (A020-2, Jiancheng, Nanjing, China) and Annexin V-FITC/PI staining (M&C Gene Technology Ltd. Beijing, China). Intracellular Fe^2+^ levels were measured using an Iron Assay Kit from Leagene. Malondialdehyde (MDA) and lipid peroxide (LPO) in kidney tissue homogenate samples and HGECs were assayed using ELISA kits (Jiancheng, Nanjing, China). Kidney tissue homogenate supernatants and the intracellular GSSG/GSH and NADP^+^/NADPH ratios were measured using glutathione and NADPH assay kits, respectively (Jiancheng, Nanjing, China).

### Metabolomics analysis

The metabolome measurements were carried out by Changzhou, China’s Zhongke Zhidian Biotechnology Co. Lipid extraction and Analyses methods based on previous reports.^79^

### Lipid peroxidation analysis using BODIPY-C11

HGECs were co-treated with 1 μM BODIPY-C11 and 1 μg/ml Hoechst for the final hour of the treatment. On a confocal laser scanning microscope (Leica, Germany), images of the reduced form of BODIPY-C11 and the oxidized form were captured at 563 nm and 488 nm, respectively. All images (14 images per well) were acquired using the same instrument specifications and processed using the same settings.

Adding 5 μM BODIPY-C11 dye to the culture medium during the final hour of the treatment. HGECs for flow cytometry analysis were trypsinized, stained with 7-AAD for 5 min, and then filtered into single-cell suspensions. PE-Texas Red filter was for reduced BODIPY-C11 and FITC filter for oxidized BODIPY-C11. With few alterations, the experimental procedure was carried out as it was in the prior report.^85^

### Phen Green SK Staining

HGECs were loaded with 10 μM Phen Green SK (PGSK, Cayman Chemical, United States) at 37 °C for 10 min after being washed with Hanks’ buffered salt solution (HBSS, pH 7.3). Hoechst 33342 was used to stain the nuclei for 10 min. The fluorescence of the images was assessed using laser scanning microscopy at an excitation wavelength of 507 nm and an emission wavelength of 532 nm. When Fe^2+^ is present, PGSK fluorescence is reduced, and the amount of reduction is inversely correlated with the concentration of Fe^2+^ in the solution.

Cell suspensions were collected and added with the PGSK probe for flow cytometry analysis. The cells were centrifuged at 300 g for 20 min after incubation, then washed and resuspended. Fluorescence activating cell sorting (FACS) was used to determine the relative PGSK level. We calculated the change in Fe^2+^ concentration using the decrease in PGSK fluorescence.

### Immunofluorescence staining

Frozen kidney slices (7 μm) were blocked for 1 h, and then incubated with primary antibody (1:50 dilution) overnight at 4 °C. Secondary antibody (1:500 dilution) was incubated on the sections for one hour at room temperature. Image J was used to analyze the scatter plot generated by colocalization to indicate the degree of colocalization. The closer the scatter plot is to the diagonal line, the higher the degree of colocalization is, and vice versa.

### Western blot analysis

In the presence of protease inhibitors, kidney tissue or HGECs were lysed. Equal amounts of protein were electrotransferred to polyvinylidene fluoride membranes after being separated by SDS-PAGE on running gels of 8%, 10%, or 12%. Treated with various antibodies (1:1000) overnight at 4 °C after being blocked with 10% bovine serum albumin, and then with secondary antibodies for 1 h at room temperature. Odyssey for infrared imaging was used to detect the immunofluorescence intensity of the bands (LI-COR Biosciences, Lincoln, NE, USA). The density of the band’s pixel intensity was measured using Image J software, and it was then normalized to the equivalent loading control intensity.

### Quantitative PCR analysis of mRNA levels

Using a reverse transcription apparatus (Promega, Madison, WI, USA), two micrograms of RNA produced with Trizol reagent (Promega, Madison, WI, USA) was converted into cDNA. SYBR Green I fluorescence and a Mx3000 Multiplex Quantitative PCR System were used to perform qPCR. The Stratagene Mx3000 software was used to calculate all the results, and the relative mRNA levels were normalized to β-actin. The primer sequences used were listed as follows: mouse *gpx4* (forward 5′-TGTGCATCCCGCGATGATT-3′; reverse 5′-CCCTGTACTTATCCAGGCAGA-3′); mouse *slc7a11* (forward 5′-AGGGCATACTCCAGAACACG-3′; reverse 5′-GGACCAAAGACCTCCAGAATG-3′); mouse *lox15* (forward 5′-GGCTCCAACAACGAGGTCTAC-3′; reverse 5′-CCCAAGGTATTCTGACACATCC-3′); mouse *acsl4* (forward 5′-CCTGAGGGGCTTGAAATTCAC-3′; reverse 5′-GTTGGTCTACTTGGAGGAACG-3′); mouse *tf* (forward 5′-GCTGTCCCTGACAAAACGGT-3′; reverse 5′-GTCACGGAAGCTGATGCACT-3′); mouse *tfr* (forward 5′-GTTTCTGCCAGCCCCTTATTAT-3′; reverse 5′-GCAAGGAAAGGATATGCAGCA-3′); mouse *slc40a1* (forward 5′-GCGATCACAATCCAAAGGGAC-3′; reverse 5′-TTGGTTAGCTGGTCAATCCTTC-3′); mouse *etnk2* (forward 5′-CGGTGGAACAGGACGACATC-3′; reverse 5′-AGGCCAATAGCTTGTTGGTGA-3′); mouse *pcyt2* (forward 5′-TGGTGCGATGGCTGCTATG-3′; reverse 5′-CCCTTATGCTTGGCAATCTCC-3′); mouse *ept1* (forward 5′-CTACTCCTGACATACTTCGACCC-3′; reverse 5′-CCACGACAATCCAAACCCAG-3′); mouse *pemt* (forward 5′-ATCACCATTGTGTTCAACCCAC-3′; reverse 5′-CCAGGGAATAGCAGGCTAGG-3′); mouse *lpcat3* (forward 5′-GACGGGGACATGGGAGAGA-3′; reverse 5′-GTAAAACAGAGCCAACGGGTAG-3′); mouse *actb* (forward 5′-GTGACGTTGACATCCGTAAAGA-3′; reverse 5′-GCCGGACTCATCGTACTCC-3′); human *ACSL4* (forward 5′-CATCCCTGGAGCAGATACTCT-3′; reverse 5′-TCACTTAGGATTTCCCTGGTCC-3′); human *LOX12* (forward 5′-ATGGCCCTCAAACGTGTTTAC-3′; reverse 5′-GCACTGGCGAACCTTCTCA-3′); human *LOX15* (forward 5′-GGGCAAGGAGACAGAACTCAA-3′; reverse 5′-CAGCGGTAACAAGGGAACCT-3′); human *GPX4* (forward 5′-GAGGCAAGACCGAAGTAAACTAC-3′; reverse 5′-CCGAACTGGTTACACGGGAA-3′); human *SLC7A11* (forward 5′-TCTCCAAAGGAGGTTACCTGC-3′; reverse 5′-AGACTCCCCTCAGTAAAGTGAC-3′); human *TFR* (forward 5′-ACCATTGTCATATACCCGGTTCA-3′; reverse 5′-CAATAGCCCAAGTAGCCAATCAT-3′); human *SLC40A1* (forward 5′-CTACTTGGGGAGATCGGATGT-3′; reverse 5′-CTGGGCCACTTTAAGTCTAGC-3′); and human *ACTB* (forward 5′-CATGTACGTTGCTATCCAGGC-3′; reverse 5′-CTCCTTAATGTCACGCACGAT-3′).

### RNA-sequencing

Con-B CM + Ang II and Hcy-B CM + Ang II treated GECs were collected, and RNA was extracted and purified. Library preparations and sequencing were conducted by BGI Gene Company (Shenzhen, China).

### Statistical analysis

GraphPad Prism 9.0 and IBM SPSS 24.0 were used to analyze the data. All results are presented as the means ± SEM with a minimum of three replicates. The Shapiro-Wilk normality test was used to determine whether the data were normally distributed. For normal distribution comparisons, Student’s *t* test or one-way ANOVA with Tukey’s analysis were performed. The significance of differences was set at *P* < 0.05.

## Supplementary information

Supplementary Materials

Table 1--Lipidomics of kidney tissue

Table 2--Free fatty acids of kidney tissue

Table 3--Oxidative phospholipidomics of GECs

## Data Availability

All data supporting this paper are presented within the paper and/or the Supplementary Materials. The datasets generated and/or analyzed during the current study are available from the corresponding author upon reasonable request. Raw data for HGEC RNA sequence was deposited in the NCBI Sequence Read Archive (SRA) database under accession number PRJNA 899819.
