# Supplementary material for: B cell-derived anti-beta 2 glycoprotein I antibody mediates hyperhomocysteinemia-aggravated hypertensive glomerular lesions by triggering ferroptosis
Source: Signal Transduct Target Ther. 2023 Mar 13;8:103. doi: 10.1038/s41392-023-01313-x (PMC10008839; doi:10.1038/s41392-023-01313-x)

Supplementary Materials for

B cell-derived anti-beta 2 glycoprotein I antibody mediates hyperhomocysteinemia-aggravated hypertensive glomerular lesions by triggering ferroptosis

Xing Du, Xiaolong Ma, Ying Tan, Fangyu Shao, Chun Li, Yang Zhao, Yutong Miao, Lulu Han, Guohui Dang, Yuwei Song, Dongmin Yang, Zhenling Deng, Yue Wang, Changtao Jiang, Wei Kong, Juan Feng*, Xian Wang*

Correspondence to: [xwang@bjmu.edu.cn&juanfeng@bjmu.edu.cn](mailto:xwang@bjmu.edu.cn&juanfeng@bjmu.edu.cn)

**This PDF file includes:**

Supplementary Fig. 1 to 4

Original Films of Western Blots

Gating strategies


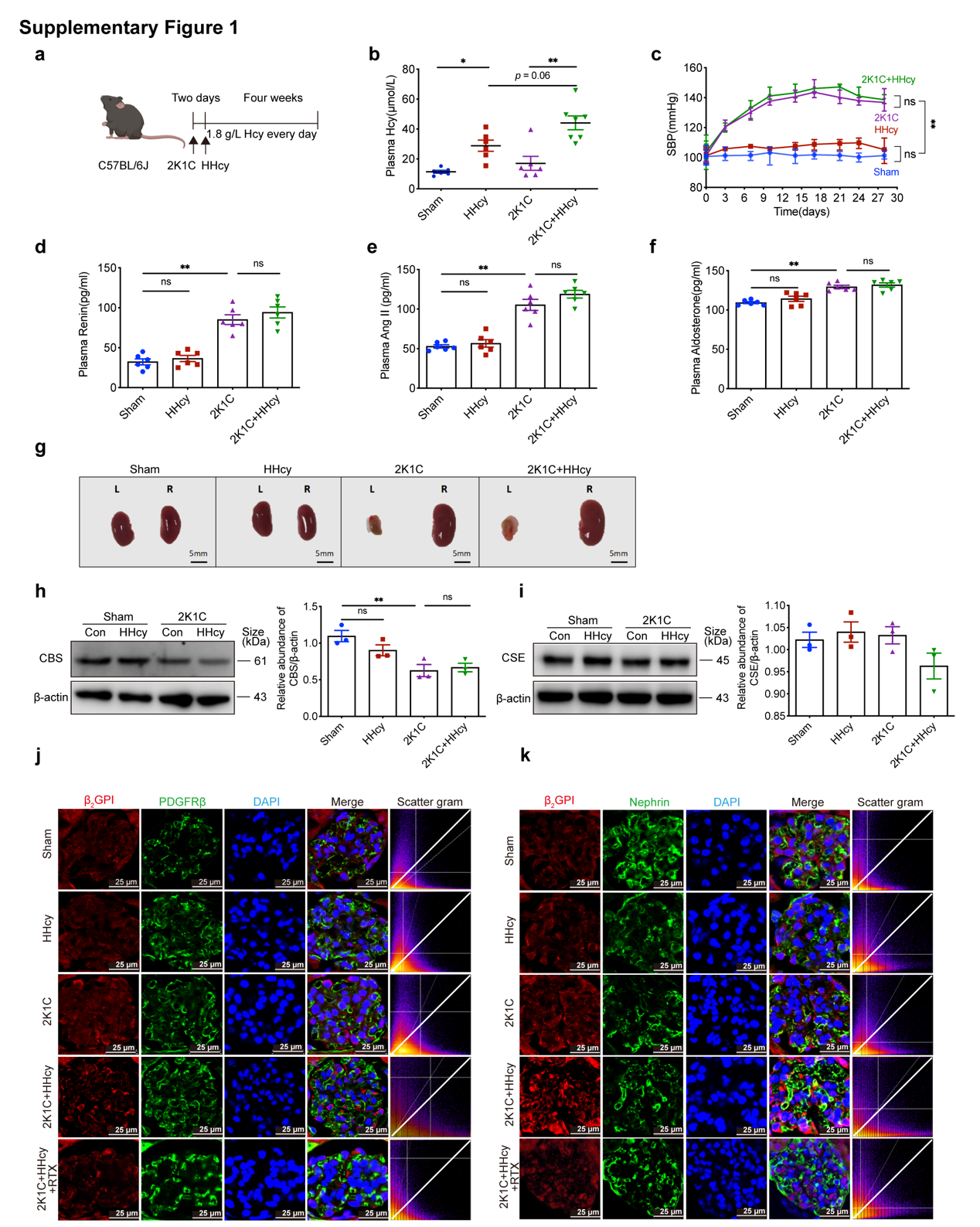


**Supplementary Fig. 1. HHcy aggravates kidney damages in 2K1C mice.**

C57BL/6J mice (8 weeks old) treated with sham or 2K1C surgery were given drinking water with or without Hcy (1.8 g/L) for 4 weeks.

(a) Schematic diagram of the strategy to generate HHcy 2K1C mice.

(b) Plasma samples were collected for the detection of Hcy levels. n = 6.

(c) Systolic blood pressure (SBP) was measured using a tail cuff on conscious mice. n = 6.

(d-f) Plasma levels of renin, Ang Ⅱ and aldosterone were assayed using ELISA. n = 6. (g) Representative images of kidneys acquired at harvesting.

(h-i) Western blot analysis of CBS and CSE protein expression in kidney tissues and quantification. β-actin was used as an internal control. n = 3.

(j-k) Representative immunofluorescent staining of β_2_GPI (red), PDGFRβ (j) (green) (scale bar, 25 μm), nephrin (k) (green) (scale bar, 10 μm) and DAPI (blue) in frozen kidney sections.

CBS, cystathionine β synthase; CSE, cystathionine gamma lyase. All data are expressed as the means ± SEM. ns, no significant difference, * *P* < 0.05, ** *P* < 0.01.


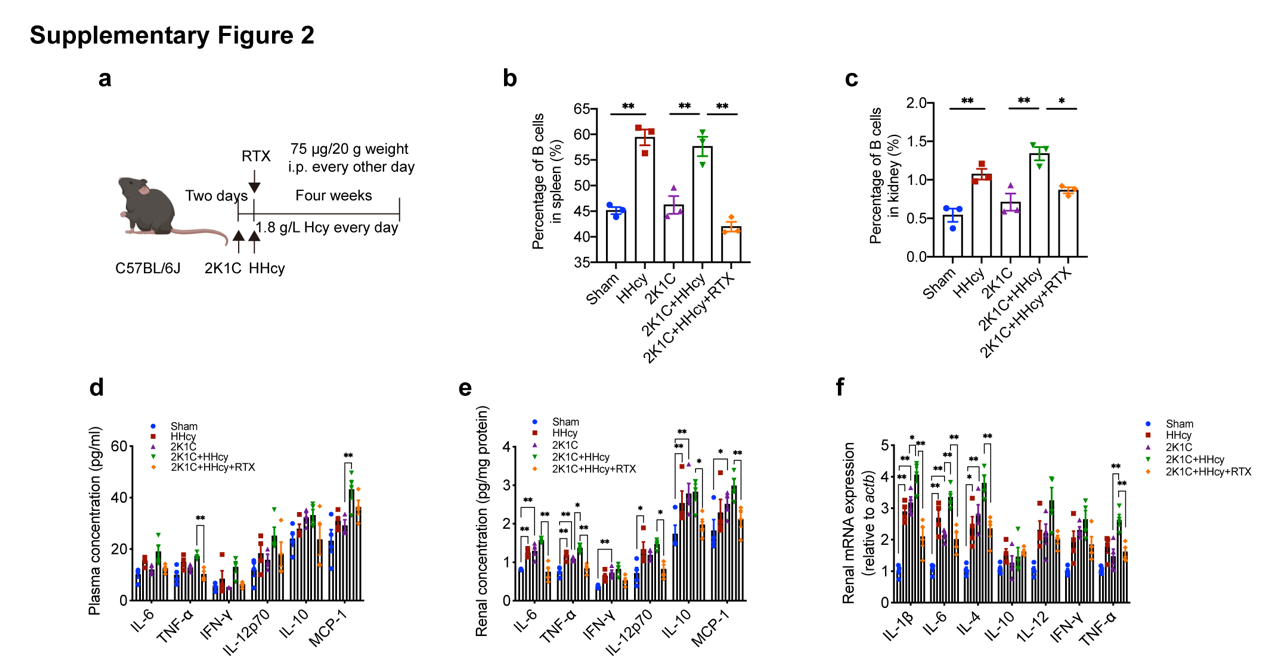


**Supplementary Fig. 2. RTX clears B cells and improves renal inflammation.**

C57BL/6J mice (8 weeks old) treated with sham or 2K1C surgery were given drinking water with or without Hcy (1.8 g/L) for 4 weeks. To assess the role of B cells in inflammation, Rituximab (RTX) was administered to HHcy 2K1C mice at the start of modeling (i.p. 75 μg/20 g body weights every other day for 4 weeks).

1. Schematic diagram of the strategy to generate 2K1C+HHcy+RTX mice.

(b-c) Percentages of B cells in spleen (b) and kidney tissue (c) were assayed using flow cytometry. n = 3.

(d) Concentrations of cytokines in the kidney were measured using a mouse inflammatory cytokine cytometric bead array (CBA) kit. n = 4.

(e) Plasma cytokines were measured using a mouse inflammatory cytokine cytometric bead array (CBA) kit. n = 4.

(f) Gene expression of inflammatory cytokines in the kidney was measured using quantitative PCR. n = 4.

All data are expressed as the means ± SEM. * *P* < 0.05, ** *P* < 0.01.


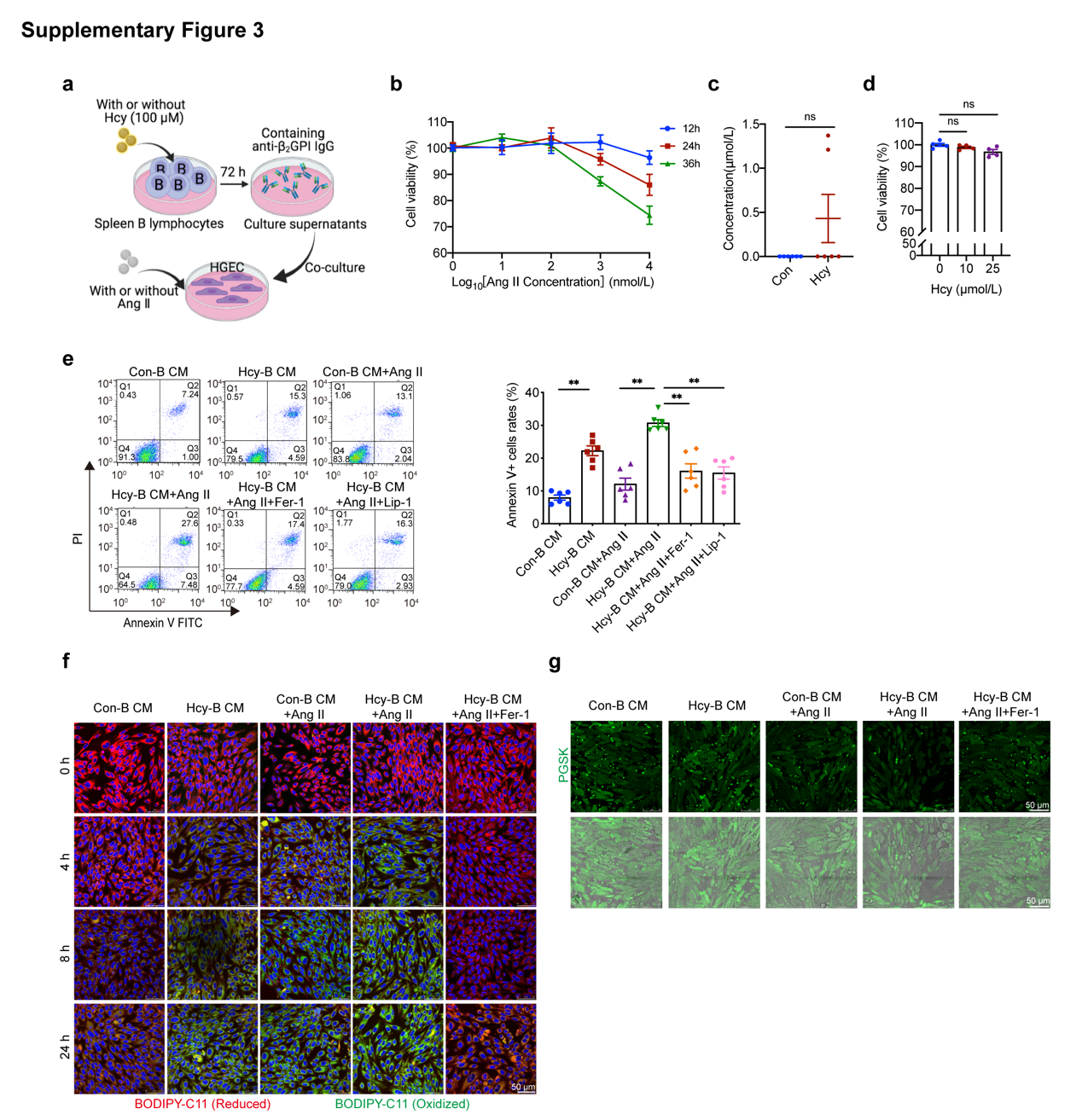


**Supplementary Fig. 3. GECs and B-cells culture supernatant co-culture *in vitro*.**

(a) Schematic diagram.

(b) Stimulation concentration and time of Ang Ⅱ were determined from the Cell Counting Kit-8 (CCK-8) assay. We chose 1 μM Ang Ⅱ and 24 h as stimulation conditions for GECs. n = 6.

(c) The Hcy levels in the culture medium of B cells treated with Hcy for 72 h were detected by ELISA. n = 6.

(d) Cell viability of GECs under 0, 10, 25 μM Hcy stimulation for 24 h were detected by CCK-8 assay. n = 4 or 5.

(e) Flow cytometry using Annexin V/PI staining was performed in GECs, and the percentages of Annexin V+ GECs were calculated in each group (ferrostatin-1, fer-1, 5 μM) (liproxstatin-1, lip-1, 200 nM). n = 6.

(f) GECs were treated as previously described for 24 h. During the last 1 h of treatment, cells were co-treated with 1 μM BODIPY-C11 and 1 μg/ml Hoechst. Images were acquired on a confocal laser scanning microscope (Leica, Germany) at 563 nm for the reduced form BODIPY-C11 and 488 nm for the oxidized form. All images (14 images per well) were collected using the same instrument parameters and processed with the same settings to maximize the ability to com-pare results between conditions. n = 6.

(g) Intracellular iron concentrations in GECs were assayed using Phen Green SK (PGSK) immunofluorescent staining. Higher Fe^2+^ concentrations are indicated by weaker PGSK fluorescence intensities. n = 6.

All data are expressed as the means ± SEM. * *P* < 0.05, ** *P* < 0.01.


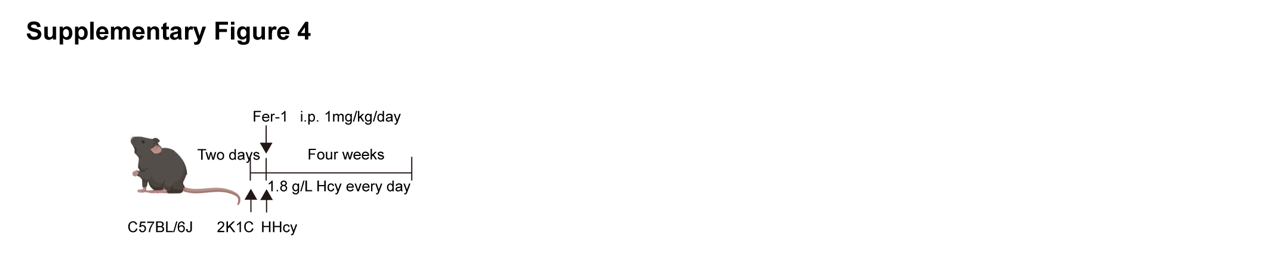


**Supplementary Fig. 4. Schematic of Fer-1 treatment in HHcy 2K1C mice.**

C57BL/6J mice (8 weeks old) treated with sham or 2K1C surgery were given drinking water with or without Hcy (1.8 g/L) for 4 weeks. Ferrostatin-1 (Fer-1) was injected into HHcy 2K1C mice (1 mg/kg/day, i.p. 4 weeks).

**Original Films of Western Blots**

**Fig. 2j**

**
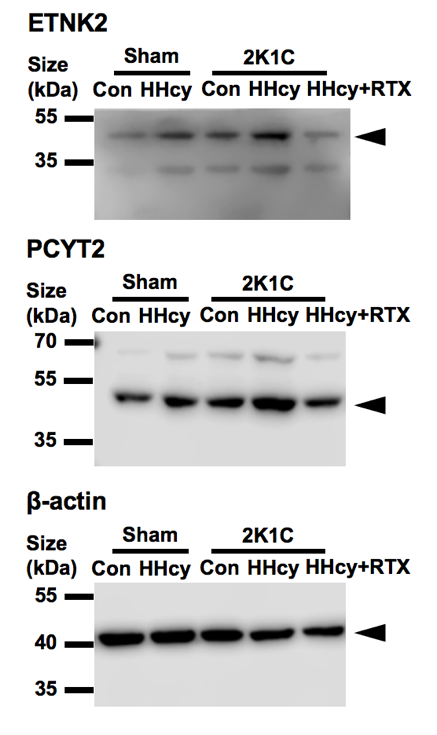
**

**Fig. 3e**


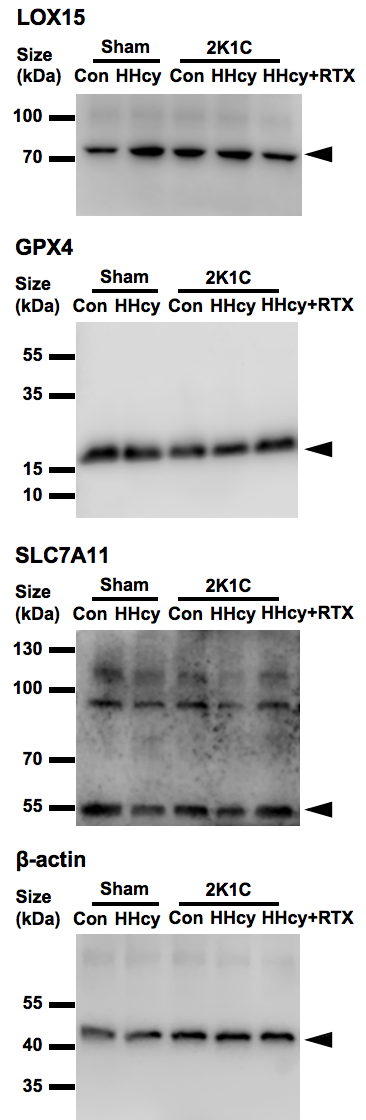


**Fig. 4e**


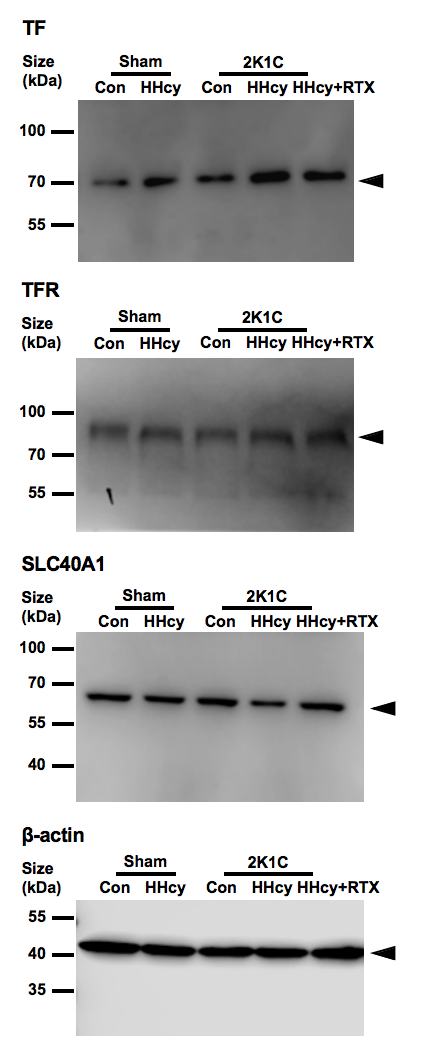


**Fig. 5d**


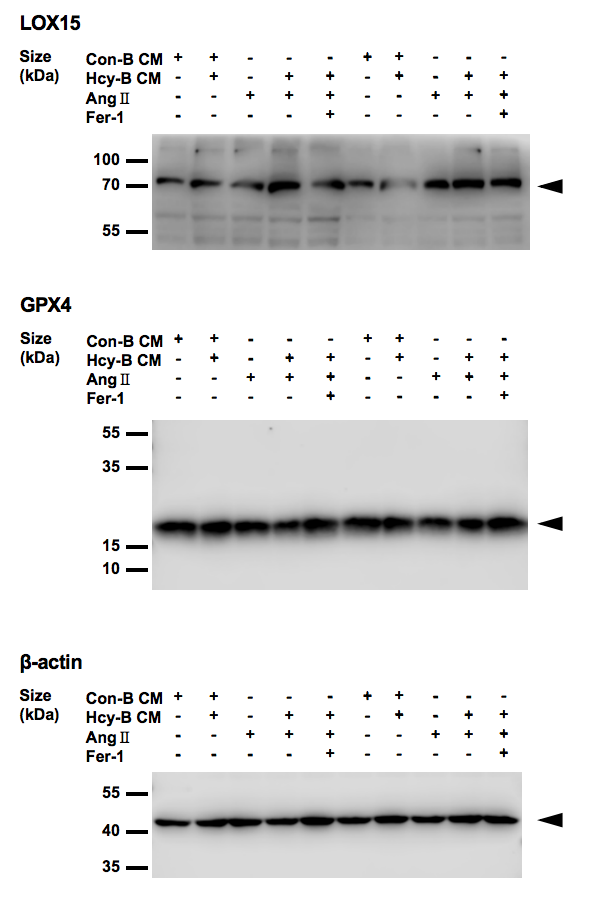


**Fig. 5k**


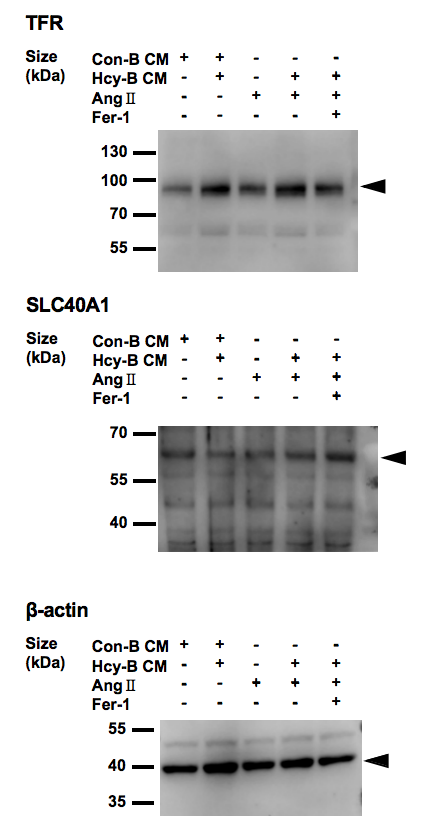


**Fig. 6c**


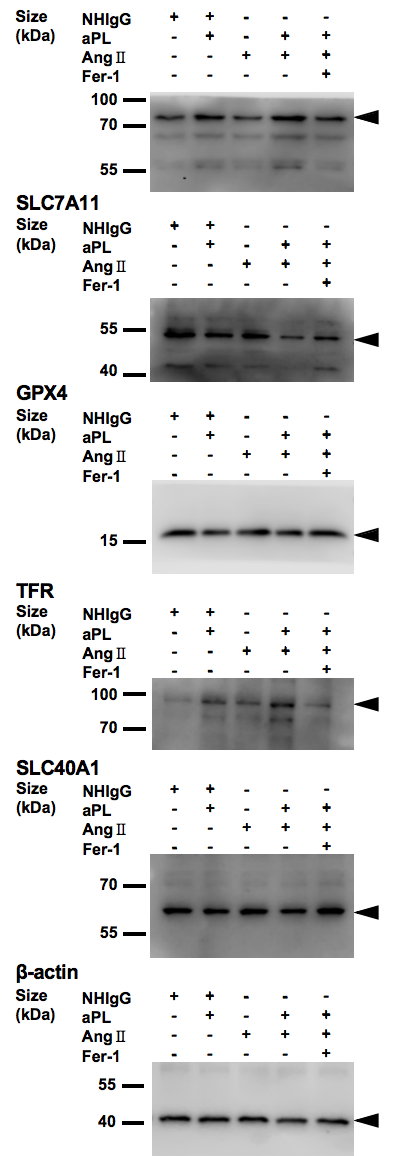


**Supplementary Fig. 1h**


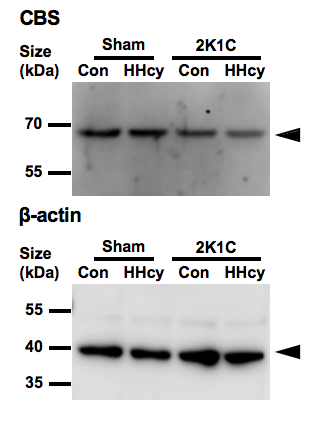


**Supplementary Fig. 1i**


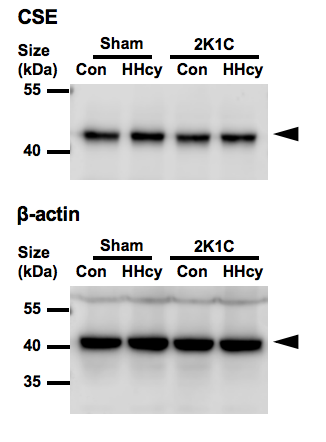


**Gating strategies**

**Fig.5g BODIPY-C11**

**
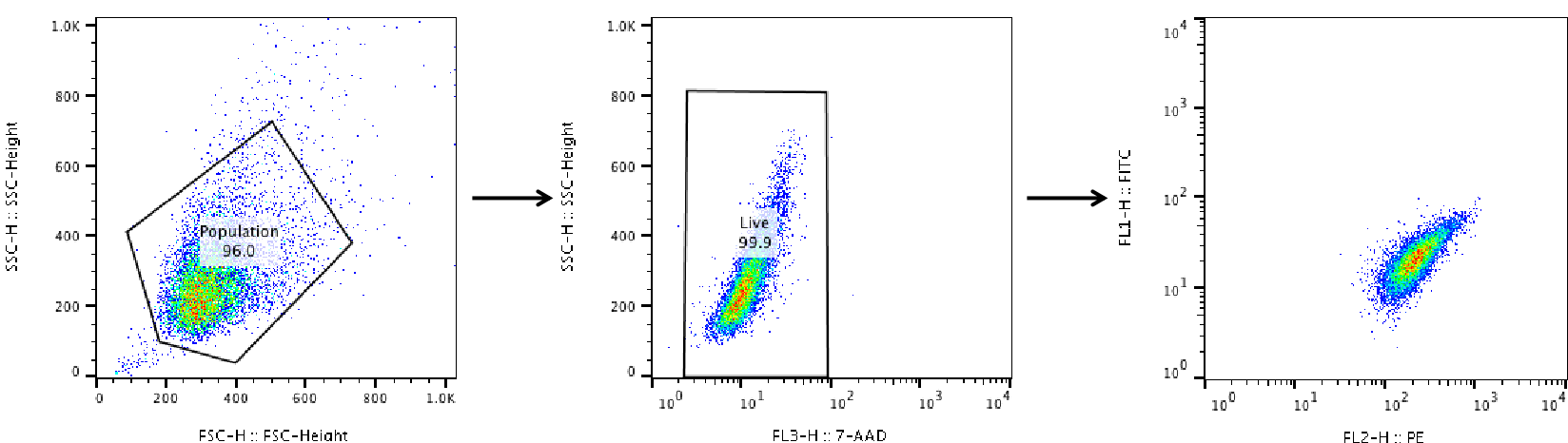
**

**Fig.5m Fig.6i PGSK**


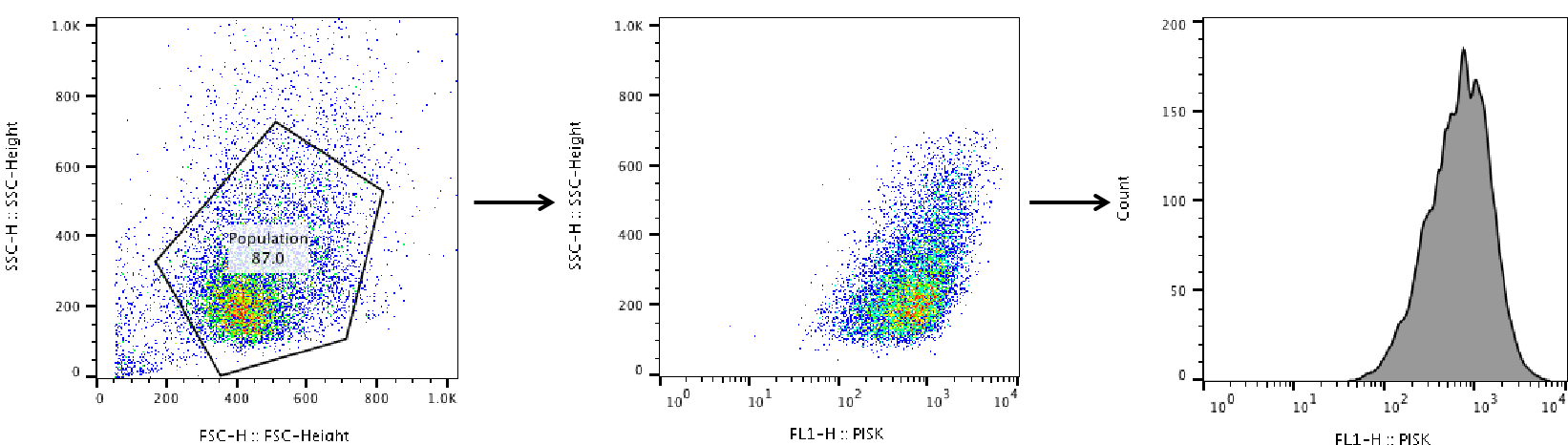

Supplement: Supplementary file 1 — Supplementary Materials [file 41392_2023_1313_MOESM1_ESM.docx]
